# Contamination by an Active Control Condition in a Randomized Exercise Trial

**DOI:** 10.1371/journal.pone.0164246

**Published:** 2016-10-10

**Authors:** Diane K. Ehlers, Jason Fanning, Elizabeth A. Awick, Arthur F. Kramer, Edward McAuley

**Affiliations:** 1 Department of Kinesiology and Community Health, University of Illinois at Urbana-Champaign, Urbana, IL, United States of America; 2 Office of the Provost, Northeastern University, Boston, MA, United States of America; Vanderbilt University, UNITED STATES

## Abstract

Contamination is commonly overlooked in randomized trials. The present study examined contamination (minutes of aerobic activity outside of exercise sessions) within an active control condition in a 6-month randomized exercise trial for older adults. We hypothesized that outside aerobic activity would be greater in the control condition compared to the intervention conditions. Participants (mean age = 65.06 years, 66.2% female) were randomly assigned to: Dance (n = 50), Walking, (n = 108), or Strength/Stretching/Stability (SSS; n = 48). Dance and Walking represented the experimental conditions and SSS the control condition. Participants attended exercise sessions three times weekly for 24 weeks. Participants recorded their physical activity outside of class on a weekly home log. Group assignment and covariates (age, gender, body mass index, exercise session intensity and enjoyment, and program adherence) were examined as predictors of weekly aerobic activity outside of exercise sessions. Participants who returned zero home logs were removed from the dataset (final N = 195). Out-of-class aerobic activity was lowest in the Walking group. Significant effects of gender, group, enjoyment, and intensity on out-of-class weekly aerobic activity were observed, all *p*<0.003. Higher perceived enjoyment of exercise sessions was associated with more out-of-class aerobic activity, while higher perceived intensity was associated with less out-of-class aerobic activity. A group x intensity interaction, *p* = 0.002, indicated that group differences in out-of-class aerobic activity were evident only among those with lower intensity perceptions. Walkers may have perceived exercise sessions as sufficient weekly exercise, while the Dance and SSS groups may have perceived the sessions as necessary, but insufficient. The lower aerobic intensity Dancers attributed to exercise sessions and non-aerobic nature of SSS may partially explain contamination observed in this study. Further examination of contamination in randomized controlled exercise trials is critically needed.

**Trial Registration:**

ClinicalTrials.gov NCT01472744

## Introduction

Although most researchers aim to maximize adherence among participants enrolled in intervention trials, minimizing contamination between conditions is an often overlooked problem in health behavior research [1,2]. Adherence refers to the degree to which the participant fulfills or completes intervention activities and is typically reported in published randomized controlled trials (RCTs). Contamination, on the other hand, reflects the extent to which participants in a control condition adopt the experimental treatment [1]. For example, in a study testing the effects of aerobic exercise on a particular health outcome, adoption of aerobic activity by the control condition may serve as a point of contamination. Designing orthogonal intervention and control groups in health behavior and psychological research is often challenging due to potential placebo effects, Hawthorne effects, attentional differences, treatment preferences, and differential outcome expectations between groups [3–7]. These biases may cause researchers to commit Type I or II errors by way of incorrectly attributing effects resulting from these biases to the active intervention or failing to observe significant effects due to control participants’ adoption of intervention-targeted behaviors [3]. Health behavior RCTs, such as exercise trials, may be particularly susceptible to contamination because participants are often unblinded to group assignment; characteristics of the groups, such as the group facilitator or social environment, may differ; and prevention of non-study activity behaviors are ethically beyond the control of the researcher [4,7–9].

While the choice of control group is ultimately determined by pilot data, research questions, and available resources, researchers have adopted a number of approaches across the phases of a behavioral treatment [4,10]. Traditional no-treatment control groups have become less common in health behavior research due to ethical considerations, and because researchers cannot ensure control group participants do not receive the experimental treatment (e.g., are not physically active) and often cannot account for other factors related to the intervention-targeted behavior (e.g., group leader characteristics, social support for physical activity). Therefore, active comparison groups may be preferred over no-treatment control groups in behavioral research [4]; yet, these designs are not without their challenges. Specifically, the introduction of a comparison health intervention may inadvertently prompt control participants to adopt behaviors similar to those targeted in the experimental condition. For example, in a trial comparing aerobic exercise, resistance training, and usual care on health outcomes in prostate cancer patients, Segal and colleagues [11] reported that 15 and 20% of participants assigned to usual care and resistance training, respectively, participated in aerobic exercise in addition to their assigned training. Despite these data, few studies have systematically evaluated contamination effects in randomized trials. Further, of those studies examining contamination, most are within the exercise oncology literature [1,2], with even fewer focusing on other populations, such as older adults. Understanding the health behaviors of participants within active comparison groups may not only help researchers better design and evaluate health outcomes studies [3,6], but may also provide valuable information to guide the design of future health behavior interventions [12].

The purpose of this study was to examine contamination in a 6-month randomized exercise trial. Contamination was operationalized as participation in aerobic activity outside of scheduled exercise sessions by the active control condition. We aimed to determine whether older adults (60–79 years-old) randomized to a non-aerobic, active control condition, Strength/Stretching/Stability (SSS), or one of two aerobic exercise conditions, Dance or Walking, differentially accrued more minutes of weekly aerobic activity outside of scheduled exercise sessions. We focused on aerobic activity because the primary targets of the active intervention conditions (Dance and Walking) were aerobic activity and fitness. We hypothesized that the SSS group would report more aerobic activity outside of schedule sessions when compared with the Dance and Walking groups. Previous research has reported associations among age, gender, body mass index (BMI), exercise attitudes, adherence, and contamination [1,7,12]. Therefore, we also explored potential covariates of contamination, including age, gender, BMI, perceived intensity and enjoyment of intervention exercise sessions, and program adherence.

## Materials and Methods

### Participants

This study was approved by the University of Illinois Institutional Review Board (IRB Protocol Number: 11454). Written informed consent was obtained from all participants. The trial was registered with United States National Institutes of Health ClinicalTrials.gov (ID: NCT01472744). Participants were 206 healthy, community-dwelling older adults (mean age = 65.06 ± 4.37 years, 66.2% female) enrolled in an RCT examining aerobic exercise training effects on cognition and brain health. Individuals were eligible to participate if they were aged 60–79 years, English speaking, right-handed, local to the study location, low-active (i.e., engaged in moderate physical activity for 20+ minutes on no more than 2 days per week over the past 6 months), willing to be randomized to one of three exercise groups, capable of participation in exercise without exacerbating preexisting conditions as determined by the personal physician, and not involved in another physical activity program. A project coordinator screened interested individuals for eligibility and enrolled eligible participants.

**Fig 1:**
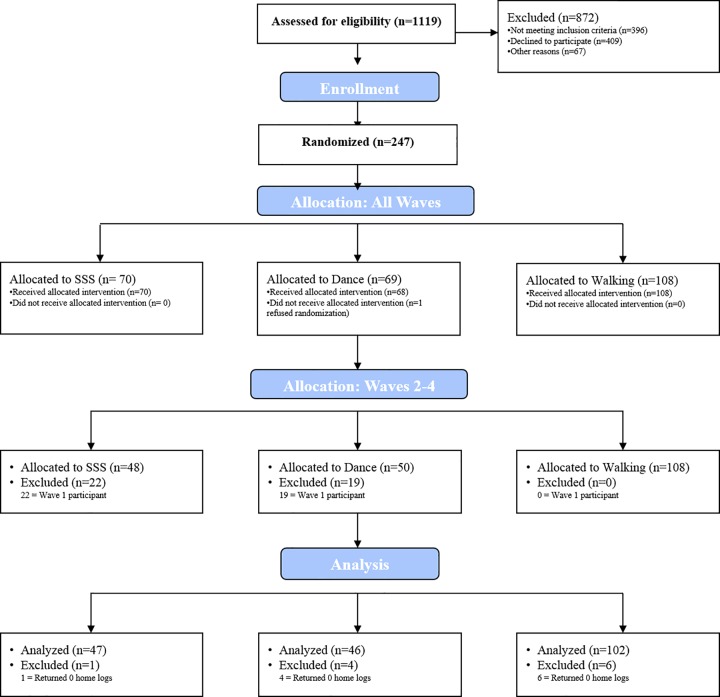
CONSORT Diagram.

Results of the present study represent secondary data collected from participants randomized to one of three exercise training interventions: Dance (n = 50), Walking (n = 108), and SSS (n = 48). The Walking group included participants assigned to Walking only and Walking plus nutritional supplement (Walking Plus). Preliminary analyses of primary outcomes indicated no differences between Walking and Walking Plus; therefore, groups were collapsed for the present analyses. The trial was powered for the primary outcomes (i.e., the effects of aerobic exercise and aerobic exercise + cognitive training on cognitive function and brain health) based upon magnetic resonance imaging data from previous studies [13–14]. Specifically, sample size was estimated for multivariate analysis of variance at a two-sided Type 1 error rate of α = 0.05 and power = 0.80. Based upon our previous studies [13–14], in order to detect a moderate effect (f = 0.25), 180 participants (n = 45 in each of four groups) were needed. After all baseline data were collected, participants were randomized using a computer data management system and baseline-adaptive randomization scheme [15]. Randomization was stratified by age in 5-year cohorts and by gender. The intervention was conducted in four waves from October 2011 to November 2014. The first wave was not included in the present analyses because the Walking group was not added until the second wave. Wave 2 recruitment began in March 2012 (intervention start: June 2012) and data collection for Wave 4 was completed in November/December 2014. The flow of participants through the trial is illustrated in Fig 1.

### Procedures

The trial was based on previous work providing consistent evidence of an association between aerobic fitness and cognitive function, brain structure, and brain function in older adults [13–14, 16–18]. Walking (aerobic exercise) and Dance (combination of aerobic exercise and cognitive training) represented the experimental conditions, while SSS (non-aerobic exercise) represented the active control condition.

All groups met three times weekly for approximately one hour over the course of 24 weeks. Across groups, each session began with a warm-up of light walking and stretching and ended with a cool-down that included stretches targeting the major muscle groups. Individuals assigned to the Dance intervention participated in social dancing comprised primarily of American and English folk dancing. Dances were led by an experienced dance instructor and were progressive in nature such that the speed, complexity, and intensity of the dances increased over each month of the intervention. The Walking intervention was also led by a trained exercise leader. Individuals assigned to Walking progressed to 40 minutes of walking at 50–60% of their maximal heart rate (as ascertained by a baseline maximal graded exercise test) during the first six weeks of the program. For the remaining 18 weeks, participants walked for 40 minutes at 60–75% of their maximal heart rate. During the SSS intervention, a trained exercise leader led participants through a series of 10–12 exercises (each session) designed to improve muscular strength and endurance, flexibility, and balance in older adults. The SSS program was designed to be progressive in that new exercises were introduced at the beginning of each month and, to increase intensity, built upon throughout the month and across the length of the intervention. No instructions or prescriptions were given to participants in any of the three groups related to their participation in exercise or physical activity outside of the study exercise sessions.

### Measures

#### Demographics

Prior to randomization, all participants completed a standard demographics questionnaire to assess their age, gender, race/ethnicity, marital status, and educational level. BMI was calculated from height and weight measured by trained research staff using a Seca stadiometer (Hamburg, Germany) at a baseline maximal graded exercise test. BMI was categorized as normal weight (BMI 18.50–24.99), overweight (BMI 25.00–29.99), and obese (BMI≥30.00).

#### Home logs

All participants were asked to record their daily out-of-class physical activity on a home log, including the type(s) of activity completed (e.g., walking, mowing the lawn, golfing with cart) each day and the duration of each bout. Logs were collected by research staff weekly during exercise sessions. Two research staff independently categorized each activity by mode and classification. Modes included aerobic, non-aerobic, combination, or unsure. Classifications included exercise, sport, leisure-time, daily living, occupational, and transportation. Staff members discussed discrepant modes and classifications until consensus was achieved. The primary outcome variable was weekly minutes of aerobic activity, which was calculated as the weekly sum of exercise, sport, and leisure-time activity minutes. We focused on these classifications of physical activity because the trial was not designed to influence participants’ occupational and transportation activity. We focused on the aerobic mode because the active interventions (Walking and Dance) targeted aerobic activity.

#### Exercise session logs

Participants recorded their overall perceived intensity and enjoyment of exercise sessions on a log at the end of each Dance, Walking, and SSS session. To rate perceived intensity, a scale including the following question was included on the exercise log: “How hard do you feel like you were working?” The perceived enjoyment scale included: “How much did you enjoy your activity session?” Perceptions were rated on a 5-point scale, with 1 corresponding with “very light” and “not at all” enjoyable and 5 corresponding with “very hard” and “very much” enjoyable. Average intensity and enjoyment across the intervention were categorized prior to analysis using median scores of average weekly perceptions (intensity = 3.68, enjoyment = 3.94).

#### Program adherence

Research staff members recorded participant attendance at each exercise session. Program adherence was calculated as the number of sessions attended divided by the total number of sessions possible.

### Data Analysis

Generalized linear mixed models in which repeated measures (n = 23) were nested within persons were used to examine differences in weekly out-of-class aerobic exercise, sport, and leisure-time activity (hereafter referred to as aerobic activity) across groups and time (N = 4485 person x time observations). We also explored exercise session enjoyment and intensity as independent predictors and potential moderators of aerobic activity outside of exercise sessions. Participants who did not return any home logs were excluded from analyses. Home log data from week 24 were also removed from analyses due to a disproportionately high rate of missingness during the last week of the intervention. Aerobic activity was positively skewed and natural log transformed prior to analysis. Hypothesized demographic covariates were tested independently prior to the primary analysis and included age, gender, BMI, and program adherence. Models were adjusted for significant demographic predictors. To account for temporal autocorrelation, an AR(1) error covariance structure was used in all models, and pairwise comparisons were tested with a Bonferroni correction. A full maximum likelihood estimation was used in all models, and model goodness-of-fit was tracked using -2 Log-Likelihood (-2LL) statistics, Akaike’s Information Criterion (AIC), and Schwarz’s Bayesian Criterion (BIC). Predictor variables were considered significant at *p* < 0.05, but were retained in the model at *p* < 0.10. A series of one-way analysis of variance (ANOVA) was conducted to describe overall group differences in covariates and predictors (i.e., BMI, exercise session intensity and enjoyment, and program adherence). All data were analyzed in SPSS 22 (Chicago, IL).

## Results

### Descriptive Measures

Table 1 provides a summary of the sample characteristics, home logs, and exercise session logs. Eleven participants did not return any home logs, leaving a final sample size of N = 195. Group assignment was not associated with removal from the dataset, *χ*^2^(2, 206) = 1.92, *p* = 0.38. No differences were observed between those included and those excluded from analyses in age, gender, BMI, and program adherence, all *p* = 0.27 to 0.96. Among those included in the analyses, Walkers returned more home logs than individuals in the Dance group, *p* = 0.02. Table 1 provides a more detailed breakdown of home log compliance across groups.

**Table 1:** Sample Characteristics (N = 195).

		Dance		SSS		Walking		Total	
		*n*	(%)	*n*	(%)	*n*	(%)	*n*	(%)
		M	± SD	M	± SD	M	± SD	M	± SD
***N* (%)**		46	(23.59)	47	(24.10)	102	(52.31)	195	(100.00)
**Female**		33	(71.74)	27	(57.45)	69	(67.65)	129	(66.15)
**Age**		65.41	± 4.28	65.21	± 4.23	64.83	± 4.49	65.06	± 4.37
**Married**		29	(63.04)	27	(57.45)	61	(59.80)	117	(60.00)
**Caucasian**		41	(89.13)	37	(78.72)	83	(81.37)	161	(82.56)
**College Graduate or more**		27	(58.70)	30	(63.83)	63	(61.76)	120	(61.54)
**Body Mass Index (kg/m**^**2**^**)**		31.21	± 5.95	31.75	± 6.30	30.46	± 4.93	30.95	± 5.53
**Home Logs Returned (N = 206)**		16.38	± 9.43	19.60	± 6.40	19.91	± 7.66	18.98	± 7.97
	0 logs returned	4	(8.00)	1	(2.08)	6	(5.56)	11	(5.34)
	1–11 logs returned	12	(24.00)	4	(8.00)	13	(12.04)	29	(14.08)
	12–16 logs returned	3	(6.00)	4	(8.33)	2	(1.85)	9	(4.37)
	17–20 logs returned	0	(0.00)	10	(20.83)	5	(4.63)	15	(7.28)
	21–23 logs returned	13	(26.00)	12	(25.00)	13	(12.04)	38	(18.45)
	24 logs returned	18	(36.00)	17	(35.42)	69	(63.89)	104	(50.49)
**Aerobic Activity**		93.04	± 82.00	101.47	± 122.55	69.70	± 115.91	82.23	± 113.87
**Exercise Session Indices**									
	**Mean Perceived Intensity**	2.93	± 0.84	3.74	± 0.55	3.83	± 0.62	3.59	± 0.76
	**Mean Perceived Enjoyment**	3.82	± 0.79	4.07	± 0.72	3.79	± 0.71	3.86	± 0.74
**Program Adherence (%)**		73.55	± 25.49	74.04	± 21.20	72.79	± 21.57	73.27	± 22.36

Note. Aerobic Activity = Average weekly minutes of self-reported aerobic leisure-time, sport, and exercise activity from the home logs

M = Mean, SD = Standard Deviation

No group differences were observed in age, gender, BMI, or program adherence, all *p* = 0.31–0.94. Group differences in perceived intensity were observed such that Dance participants perceived exercise sessions as lower intensity compared with the Walking and SSS groups, both *p* < 0.001. Group differences in enjoyment were marginal, *p* = 0.086, with the Walking group reporting slightly less enjoyment of exercise sessions than the SSS group. Examination of enjoyment and intensity categories revealed similar results. While the median split for enjoyment yielded similar distributions across groups, a significantly greater number of Dance participants perceived their exercise sessions as lower than the median intensity (82.6%) when compared with the other two groups, *χ*^2^(2, 194) = 26.98, *p* < 0.001.

### Home log derived estimates of weekly aerobic activity outside of exercise sessions

**Fig 2:**
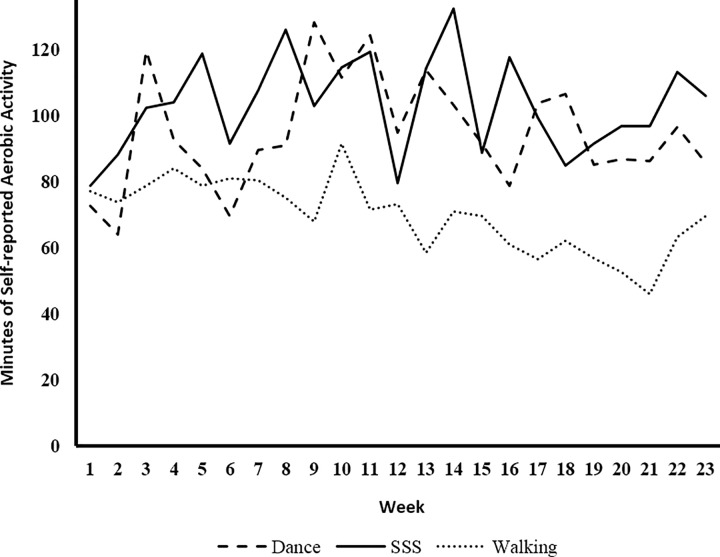
Weekly Aerobic Activity Outside of Program Sessions.

Results of the linear mixed models are reported in Table 2. Age, BMI, and program adherence were not significantly associated with weekly out-of-class aerobic activity (all *p* > 0.46). A main effect of gender was observed such that females reported more weekly aerobic activity outside of class across groups and time, *p* < 0.001. All subsequent models were adjusted for gender. A significant group effect was observed in which the Walking group reporting fewer minutes of out-of-class aerobic activity across the intervention than the Dance and SSS Groups, *p* = 0.009, and < 0.001, respectively. Although no group x time effect was evident, *p* = 0.43, the univariate test of the interaction revealed significant decreases in out-of-class aerobic activity in the Walking group, *p* = 0.036, with activity levels remaining stable in the Dance, *p* = 0.50, and SSS, *p* = 0.64, groups over time (Fig 2).

**Table 2:** Fixed Effects on Weekly Aerobic Activity.

	Numerator df	Denominator df	*F*-statistic	*p*-value
Intercept	1	541.49	408.45	<0.001
Time	22	2269.29	1.43	0.09
Gender	1	550.94	12.44	<0.001
Group	2	555.48	13.85	<0.001
Exercise Session Enjoyment	1	553.76	7.91	0.005
Exercise Session Intensity	1	556.93	13.34	<0.001
Exercise Session Intensity x Group	2	554.30	6.47	0.002

-2 Log-Likelihood: 15238.31

Akaike’s Information Criterion: 15302.31

Schwarz’s Bayesian Criterion: 15501.69

**Fig 3:**
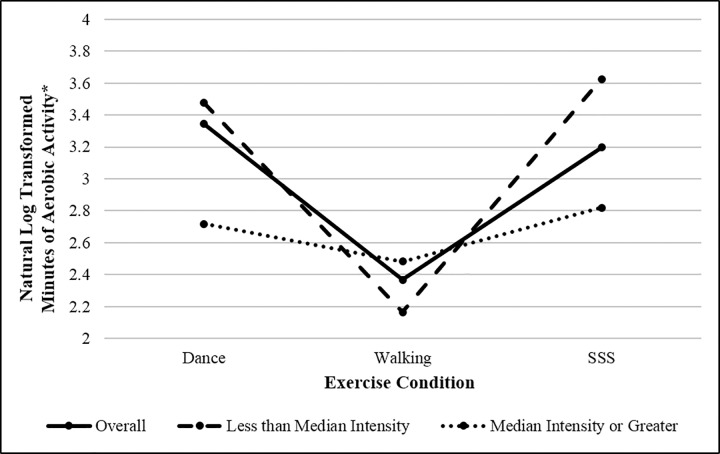
Group differences in out-of-class aerobic activity overall and by perceived intensity of exercise sessions. *Mean unstandardized predicted values (adjusted for gender).

Significant main effects of perceived enjoyment and intensity (*p* = 0.005 and < 0.001, respectively) were observed in relation to out-of-class aerobic activity, with higher perceptions of enjoyment associated with more out-of-class aerobic activity across the intervention and higher perceptions of intensity associated with lower out-of-class aerobic activity. A group x intensity interaction was also observed, *p* = 0.002. Pairwise comparisons indicated the main effect of group was driven by differences in out-of-class aerobic activity within individuals reporting lower than the median perceived intensity across the intervention, *F*(2,558.31) = 19.50, *p* < 0.001 (Fig 3). Specifically, Walkers who perceived their sessions as lower intensity reported less out-of-class aerobic activity across the intervention when compared with participants in both the Dance and SSS groups who also perceived their sessions as lower intensity, both *p* < 0.001. Examination of the interaction by group also indicated that participants in the Dance and SSS groups reported more out-of-class activity if their perceived intensity of exercise sessions was lower than the median, *F*(1,558.06) = 6.60, *p* = 0.01 and *F*(1,556.67) = 13.19, *p* < 0.001, respectively.

## Discussion

The purpose of this study was to examine the contamination by an active, non-aerobic comparison group in a randomized exercise trial. Specifically, we examined group differences in self-reported aerobic activity outside of scheduled exercise sessions. Out-of-class aerobic activity decreased in the Walking group across the intervention, but was maintained in Dance and SSS (Fig 2). Major findings suggest that exercise mode and perceived intensity may have contributed to participants’ weekly activity outside of exercise sessions. The Walking group may have perceived the three-times weekly walking sessions as sufficient, while the Dance and SSS groups may have perceived the three-times weekly exercise sessions as a necessary, but insufficient amount of weekly exercise, particularly if perceptions of intensity were low. While enjoyment of exercise sessions emerged as a significant predictor of out-of-class aerobic activity across groups, intensity was the stronger predictor and may have been responsible for group differences observed. Traditional pre-post measures of physical activity may not be adequate for examining contamination effects. The weekly home logs, on the other hand, may provide more comprehensive insight into activity behaviors across conditions.

Contamination observed in this study was not driven by major increases in aerobic activity in the control condition (i.e., SSS), but by decreases in out-of-class aerobic activity in one of the aerobic conditions (i.e., Walking). These results are similar to a previous study in which participants assigned to aerobic conditioning training demonstrated decreases in objectively measured physical activity outside of scheduled sessions [19]. However, findings are also contrary to the results of a review of exercise oncology trials by Steins Biscchop and colleagues [2] in which contamination was lowest in trials that included a concurrently implemented alternative intervention as the control condition. Despite the recommendation to incorporate a concurrent alternative intervention to minimize contamination in randomized exercise trials, the authors recognized that the optimum type of alternative intervention requires further exploration. Findings from our study indicate that a non-aerobic active intervention may not succeed in minimizing contamination in trials examining the effects of aerobic fitness on health outcomes. Other studies further support the present findings, suggesting that contamination may not be due to potential improvements in aerobic fitness as a result of non-aerobic exercise, such as resistance training, but to engagement in aerobic activity outside of scheduled sessions by control participants [11]. Participants in the current study were not provided any instruction relative to their participation in physical activity or exercise outside of scheduled sessions. SSS members’ mere participation in a non-aerobic exercise intervention may have encouraged their adoption of aerobic activity outside of scheduled exercise sessions. To better test contamination, future efficacy trials may aim to blind participants to other exercise conditions and specifically instruct participants not to engage in physical activity outside of the intervention [7]. This may strengthen the evidence resulting from health outcomes RCTs and improve both the dissemination of evidence-based interventions and recommendations for enhancing aspects of health, such as cognitive function.

Perceived intensity and enjoyment of exercise sessions may also help to explain group differences in out-of-class activity. Despite the aerobic nature of the Dance program, participant intensity perceptions suggest Dance sessions may not have successfully engaged participants in moderate-to-vigorous physical activity as intended. This was illustrated by lower mean perceptions of intensity among Dancers, in addition to a skewed distribution of Dancers around the sample median intensity. Further, the interaction effect indicated that, while Walkers’ out-of-class aerobic activity was not dependent upon their perceived intensity of exercise sessions, differences in out-of-class aerobic activity were observed in the Dance and SSS groups by intensity category. Given the distribution of intensity perceptions within the Dance group (i.e., 82.5% below the median), intensity may specifically explain Dancers’ maintenance of aerobic activity outside of exercise sessions. While these results do not present any threats of contamination, they do suggest the Dance program may not have effectively exposed participants to adequate amounts of moderate-to-vigorous intensity physical activity. In addition to contamination, this information may be critical when explaining the study’s primary outcomes [3].

Participants who perceived their exercise sessions as enjoyable were also more likely to participate in aerobic activity outside of exercise sessions. SSS participants reported marginally higher levels of program enjoyment across the intervention when compared with the Walking group. As such, enjoyment may have further contributed to contamination behaviors observed in this study. Further research to identify additional drivers of out-of-class activity in aerobic exercise programs is needed, not only to better explain primary findings in randomized exercise trials, but also to identify important predictors of adherence and contamination [12]. For example, McAuley and colleagues [20] found that baseline levels of task coordination, inhibition, and self-regulatory strategy use predicted participant adherence to a 12-month exercise intervention indirectly through self-efficacy. Additionally, participant perceptions of social interaction with the group during exercise sessions, the exercise leader’s personality, and personal bonds with the exercise leader have been identified as important determinants of program adherence [8, 9]. Despite these findings, investigation of behavioral skills, perceptions, expectations of exercise programs, and the social-environmental and instructor characteristics of programs as determinants of exercise outside of program sessions is lacking in the physical activity literature. Such an examination may be useful in reducing contamination in RCTs and in designing future interventions targeting participants differentially based upon baseline characteristics identified.

Overall, results suggest the potential need for systematic changes in the way researchers design and analyze RCTs. Maintenance of out-of-class aerobic activity in the SSS group and decreased levels of out-of-class aerobic activity in the Walking group may represent unintended confounders of the primary relationships tested in the present RCT (i.e., effects of aerobic fitness training [Walking] and combined aerobic fitness/cognitive training [Dance] on cognition and brain structure and function). Therefore, in addition to measures of program adherence in intervention groups, measures of contamination in control groups should be considered when evaluating the efficacy of health behavior RCTs [2]. Additionally, investigations of the determinants of contamination may help to reduce the threat to internal validity in subsequent RCTs. For example, in an RCT with colorectal cancer survivors, Courneya and colleagues [12] found that baseline intentions for exercise significantly predicted contamination in the control group. Results of the present study suggest analysis of process measures, such as perceived exercise session intensity and enjoyment, in addition to baseline participant characteristics, may be needed. Such evaluation may help researchers to better explain primary outcomes, identify groups for which health behavior interventions are optimized, and design high-fidelity interventions.

This study is one of the few to empirically and longitudinally investigate the extent to which participants assigned to an active comparison adopted the experimental treatment outside of scheduled sessions. A major strength of this study is the inclusion of weekly out-of-class activity data across a 6-month exercise trial. However, this study is not without limitations. First, as the home log data relied upon participant self-report, weekly estimates of aerobic activity were vulnerable to known biases of self-report [21–23]. Additionally, while home log compliance was high overall (median = 24 home logs), a considerable number of participants (n = 40) returned fewer than 50 percent of home logs, with a disproportionate number assigned to the Dance group (Table 1). Future research employing ongoing, objective measures of physical activity may limit issues related to response bias and compliance and contribute important information on contamination in exercise RCTs. Commercially available technologies may provide a promising alternative or supplement to weekly self-report, as many are small and comfortable; provide wireless, real-time access to participant data; have reasonably large battery and memory capacities and are easily chargeable and downloadable; and are undergoing testing in laboratory and free-living environments [24]. When calculating weekly aerobic activity from the home logs, an inclusion criterion related to the perceived intensity of daily exercise, leisure-time, and sport activity bouts was not applied. Therefore, home log-derived estimates of aerobic activity may have included light intensity activity as well as moderate-to-vigorous intensity activity. As such, the observed group differences in out-of-class aerobic activity may reflect greater lifestyle activity among participants in the Dance and SSS groups as compared to the Walking group. Finally, consistent with previous research, contamination was operationalized as adoption of aerobic activity outside of exercise sessions by the active control group (i.e., SSS) [1–2]. However, other factors, such as attitudes towards exercise [10], increased awareness of physical activity, or enrollment in other exercise programs by participants in any condition, could serve to contaminate the effects of the active intervention.

## Conclusions

In summary, the findings contribute to the scientific discussion regarding the balance between rigor and ethics in designing appropriate comparison groups. Research on the contamination effects of control groups is noticeably absent from the literature, yet represents an important area of study for advancing our knowledge about physical activity’s contribution to a variety of health outcomes. Findings suggest that moderate-to-vigorous intensity aerobic interventions may result in perceptions of sufficiency, leading participants to engage in less aerobic activity outside of scheduled sessions. Participants assigned to non-aerobic or lower intensity aerobic interventions, on the other hand, may view exercise sessions as necessary, but not sufficient. Further examination of contamination effects in randomized controlled exercise trials is critically needed.

## Ethical Approval

This study was approved by the University of Illinois at Urbana-Champaign Institutional Review Board. All procedures performed were in accordance with the ethical standards of the institutional review board and with the 1964 Helsinki declaration and its later amendments.

## Supporting Information

S1 File Contamination Dataset. (CSV)

S2 File IRB application (3-21-2012). (PDF)

S3 File CONSORT Checklist. (DOC)
