# Supplementary material for: Contamination by an Active Control Condition in a Randomized Exercise Trial
Source: PLoS One. 2016 Oct 10;11(10):e0164246. doi: 10.1371/journal.pone.0164246 (PMC5056678; doi:10.1371/journal.pone.0164246)
Supplement: S2 File — (PDF) [file pone.0164246.s002.pdf]

University of Illinois
at Urbana–Champaign

Institutional Review Board Office
Suite 203, MC-419
528 East Green Street
Phone: 217.333.2670
Fax: 217. 333.0405
E-mail: irb@uiuc.edu
Web: www.irb.uiuc.edu

Research Amendment

For Submitting Changes to Previously Approved Human Subjects Research

All applications must be completed, signed by the RPI, and submitted either electronically or single-sided hard copy. Please - No Staples

Form Version 1.05

All modifications to human subjects research must be reviewed and approved prior to implementation.

Minor modifications Minor modifications to previously approved projects include those that do not alter the risk–benefit assessment for the research. Examples include changes in the investigators; minor changes in the consent form(s), recruiting materials, measures, or procedures; minor changes in compensation, time of participation, or subject recruitment; or the use of a new site that is not materially different from a previously approved site. Minor modifications may also include changes to other parameters, whereby the investigator provides the subjects with more accurate information as a result of additional experience with the protocol.

Major modifications Major modifications include significant protocol changes that would cause subjects to engage in activities not previously approved; or that involve an increased level of risk to the physical, emotional, or psychological well-being of participants (including the loss of confidentiality); or that involve a decreased benefit; or that otherwise result in alteration of the risk–benefit assessment for the research. For example, adding a new subject population, adding new measures that significantly differ from those currently approved, changing inclusion or exclusion criteria, changing the informed consent process, and changing procedures affecting subject confidentiality are all potentially major modifications.

1. DATE THIS REPORT WAS COMPLETED: 2/15/2012 **AMENDMENT NUMBER (START WITH 01):** 04

2. RESPONSIBLE PROJECT INVESTIGATOR (RPI) AT UIUC

Last Name: Kramer	First Name: Arthur	Academic Degree(s): Ph.D.
Dept. or Unit: Beckman Institute	Office Address: 1st floor directors offic	Mail Code: 251
Street Address: 405 N. Mathew	City: Urbana	State: IL Zip Code: 61801
Phone: 217-244-8373	Fax:	E-mail: a-kramer@illinois.edu
UIUC Status: Non-visiting member of (Mark One) ☒ Faculty ☐ Staff		

3. PROJECT TITLE **IRB PROTOCOL NUMBER:** 11454

Influence of Fitness on Brain and Cognition II

4. MAJOR OR MINOR MODIFICATION? In the RPI's judgment, which category of modification is this?

☐ Minor ☒ Major ☐ Uncertain

5. REVISED MATERIALS: For revisions to currently approved procedures (including discontinuation of previously approved procedures, measures, etc.), or to add new procedures that were not previously approved, please resubmit the IRB-1 or Application for Exemption incorporating the revisions as appropriate throughout the form. Amendments often require modification of consent forms, assent forms, measures and other relevant attachments.

PLEASE SUPPLY THE FOLLOWING with this Research Amendment:

a) A marked up version of the IRB-1 Application or Application for Exemption and any modified attachments or consent documents. NOTE: If your computer does not allow "strike-throughs" or other editing on the IRB-1 or Application for Exemption, it is acceptable to cross off deleted sections with a pen and use a highlighter to emphasize changes.
b) The **entire** IRB-1 Application or Application for Exemption reflecting the revisions.
c) Revised consent documents and other relevant attachments that have changed as a result of the amendment

→ Mark One: Changes marked versions and final versions are: ☒ Attached ☐ Will Follow.

6. DESCRIBE THE AMENDMENT. Describe the requested change(s) and clearly reference materials submitted with this form. Provide a clear rationale for the proposed change(s). Explain whether the risk–benefit assessment for the research is likely to change as a result of the proposed amendment(s). Justify changes that will affect risks, benefits, informed consent, inclusion or exclusion criteria, the subject population(s), research sites, or the confidentiality of private, identifiable subject information.

This amendment addresses the addition of funding for this project (IRB-1, #5B) through the Center for Nutrition, Learning, and Memory, which will incorporate additional measures and interventions as described here. These changes are only applied to the group of participants labeled as non-active and do not affect the ACTIVE group nor any of the documents and protocols associated with that group.

1. Two new intervention arms will be added: a traditional walking program, which will have participants walking around a dedicated track, and a traditional walking program plus a nutritional supplement to be provided by Abbott Nutrition. (IRB-1, #7)

2. Participants will be asked to provide a blood sample that will be collected in Dr. Jeffrey Woods' lab (IRB-1, #8). They will provide a blood sample for the following analysis: C-reactive protein (CRP), Interleukin-6 (IL-6) – proteins found in the blood that respond to inflammation; brain-derived neurotrophic factor (BDNF) – a secreted protein that is related to brain function in the hippocampus, cortex and basal forebrain, areas related to learning, memory and higher thinking. The purpose of these analyses is to determine any changes following the six-month physical activity interventions.

3. Additional co-principal investigators have been added: Dr. Jeffrey Woods, Dr. George Fahey, as well as additional staff. (IRB-1, #3, #4)

4. Participants in the walking intervention will be provided with Heart Rate Monitors. (IRB-1, #13)

5. Total number of participants was increased to accommodate the new intervention arms. (IRB-1, 9C)

6. Divided the questionnaires (into three packets instead of two, adding a C-packet) to alleviate participant burden. (IRB-1, #16)

7. Changed the inclusion/exclusion criteria due to the blood analysis. (IRB-1, #11)

8. A questionnaire had been inadvertently left off of the list that was reformatted with the addtion of the C-packet. CES-D (#21) was added back in. (In the original IRB-1.)

If additional Item 6 information is attached, check here: ☐

7. INVESTIGATOR ASSURANCES The original, inked signature of the Responsible Project Investigator is required before this form can be processed. Other investigators are also responsible for these assurances and are encouraged to sign. Neither stamps nor proxy signatures are accepted in this section.

I certify that the information supplied in this form, with attachments, is complete and correct, that the modified protocol has not yet been used with any human subject, and that it will not be implemented until IRB approval has been obtained.

<u>NOTE:</u> The signature of the RPI must be submitted before IRB Review (scanned or faxed signatures are acceptable).

Responsible Principal investigator	Date	Investigator	Date
[signature]	3/21/12		
Investigator	Date	Investigator	Date

University of Illinois
at Urbana–Champaign

Institutional Review Board Office
Suite 203, MC– 419
528 East Green Street
Phone: 217.333.2670
Fax: 217. 333.0405
E-mail: irb@uiuc.edu
Web: www.irb.uiuc.edu

Biological Materials Form—Appendix C

For the Collection, Analysis, or Banking of Human Origin Materials

1. **INSTRUCTIONS** **All forms must be typewritten.** Researchers planning to collect, analyze, or bank human cells, tissues, fluids, DNA, or other human biological samples, whether taken prospectively or retrospectively with regard to IRB approval, must complete this form and include it with an IRB application.

2. **RESPONSIBLE PROJECT INVESTIGATOR (RPI)** Form Version 1.02

Last Name: Kramer	First Name: Arthur

3. **PROJECT TITLE**

Influence of Fitness on Brain and Cognition II

4. **DESCRIBE THE MATERIALS** Describe the materials that will be collected, analyzed, or banked by this investigator.

Blood will be drawn from participants who have signed the Informed Consent for the study known as FAST, IRB# 11454.

List additional Item 4 information on an attachment and check here: ☐

5. **ANSWER EACH QUESTION BELOW**

5a. How and from where (name the entity) or from whom (describe the subject population) will the samples be obtained?

See attached.

List additional Item 5a information on an attachment and check here: ☒

5b. Check all that apply:

☐ Samples are **unidentified**—identifying information[1] was not or will not be collected or, if collected by a repository, was not maintained and cannot be retrieved by the repository.
☒ Samples are **identified**—links to identifying personal information exist somewhere or will be collected and maintained.

If identified,
- ☐ Samples are **unlinked**—provided to the investigator **without identifiers or codes** that can link to identifiers.
- ☒ Samples are **coded**—provided to the investigator **with codes** that are linked to identifiers.
- ☐ Samples are **identified**—provided to the investigator **with identifying personal information**.

5c. What is the intended use of the samples for purposes of the current protocol?

See attached.

List additional Item 5c information on an attachment and check here: ☐

5d. Will the samples be destroyed after this purpose is served? ☒ Yes ☐ No

If No, answer questions 5(d)(1) through 5(d)(4), on the next page.

[1] Identifying information is personal information that could be used to identify the donor by name, ID or patient number, or clear pedigree (relationship to a family member whose identity is known).

5d(1) How will the samples be stored?

Samples will be frozen and stored in a -80 C freezer.

List additional Item 5d(1) information on an attachment and check here: ☐

5d(2) Where will the samples be stored?

Samples will be stored in the Exercise Immunology Research Laboratory in Freer Hall.

List additional Item 5d(2) information on an attachment and check here: ☐

5d(3) How long will the samples be stored?

Samples will be stored until final blood draw analysis is complete.

List additional Item 5d(3) information on an attachment and check here: ☐

5d(4) Will additional purposes be devised for these samples over the long term? ☐ Yes ☒ No If Yes, explain:

List additional Item 5d(4) information on an attachment and check here: ☐

5e. Will any subject receive information from the analysis of their sample(s)? ☐ Yes ☒ No If Yes, explain:

List additional Item 5e information on an attachment and check here: ☐

5f. Who will "own" the samples and the data derived from them?

University of Illinois and the research team associated with this project.

List additional Item 5f information on an attachment and check here: ☐

5g. Will the investigator of this research remain in control of the samples and data? ☒ Yes ☐ No If No, explain:

List additional Item 5g information on an attachment and check here: ☐

5h. Will the samples or data be shared with other UIUC investigators? ☐ Yes ☒ No If Yes, explain how and with whom:

List additional Item 5h information on an attachment and check here: ☐

5i. Will the samples or data be shared with outside of UIUC? ☒ Yes ☐ No If Yes, explain how and with whom:

Co-investigator Michelle Voss, University of Iowa, will have access to de-identified blood data information in the form of an SPSS database.

List additional Item 5i information on an attachment and check here: ☐

5j. Will the subjects have the option of specifying future use or non-use of the samples? ☐ Yes ☒ No

5k. Is all of this information clearly explained in the relevant consent form(s)? ☒ Yes ☐ No

Biological materials From – Appendix C

Additions

5a. These blood drawing appointments will be held in the Exercise Immunology Research Laboratory located in Freer Hall, room 125 on the UIUC campus. A trained phlebotomist will draw blood samples from participants volunteering for the project known as FAST. These participants are between the ages of 60-79 years of age and meet the inclusion requirements for this study.

5c. The blood will be analysed for: C-reactive protein (CRP), Interleukin-6 (IL-6) – proteins found in the blood that respond to inflammation; brain-derived neurotrophic factor (BDNF) – a secreted protein that is related to brain function in the hippocampus, cortex and basal forebrain, areas related to learning, memory and higher thinking. All analyses will take place in the Exercise Immunology Research Laboratory located in Freer Hall, room 125 on the UIUC campus. The purpose of these analyses is to determine the differences and/or changes that occur after participating in the four arms of interventions (dance group; stretching, strength, stability group; walking group; walking plus nutritional supplement group)

UNIVERSITY OF ILLINOIS
AT URBANA-CHAMPAIGN

Department of Kinesiology and Community Health
College of Applied Health Sciences
129 Huff Hall, MC-588
1206 South Fourth Street
Champaign, IL 61820 USA

Name ______________________
[Please print]

Fit & Active Seniors Trial (FAST)

at the University of Illinois at Urbana-Champaign

PARTICIPANT CONSENT FORM FOR BLOOD COLLECTION

Description

In this study we are asking you to donate a small amount of blood. We will be drawing a small blood sample and will give you instructions on how to prepare for this session in advance (e.g. overnight fasting). The maximal amount of blood drawn during any single sampling will be no more than 40mL (about 2.5 tablespoons), which is much less than that taken during one donation at the Red Cross Center or a blood drive (500 mL). The procedure will take approximately 10 minutes.

We will examine growth factors, inflammatory markers and oxidative stress levels in the blood. Specifically, these tests are:

- C-reactive protein [CRP] and Interleukin-6 [IL-6] which are proteins found in the blood that respond to inflammation levels in the body;
- Brain-derived neurotrophic factor (BDNF), a secreted protein that is related to brain function in the hippocampus, cortex and basal forebrain, areas related to learning, memory and higher thinking.

These measurements will be taken before you begin the FAST program and repeated six months later, at the end of the program. These blood samples will be stored frozen in the Exercise Immunology Research Laboratory in Freer Hall on the University of Illinois campus until the final analyses are complete. They will then be destroyed according to standard University protocol.

Risks

The blood donation procedure is very common and involves minimal risk. There is a one in five chance of bruising in the area of sampling. This is generally not serious and will completely disappear within several days. As with all invasive procedures there is a slight risk of inflammation and infection. This risk will be minimized by the use of sterile procedures and equipment at all times. There is also a possibility of dizziness and lightheadedness associated with blood draws. However, you will be seated or lying down during and immediately following the blood draw, which will reduce the possibility of injury from a fall. These risks will also be minimized because a trained phlebotomist will draw all blood samples.

Benefits

There is no medical benefit to individuals who take part in this aspect of the study. We do, however, anticipate that participation in this project will contribute further to our understanding of the relation between physical, psychological, and mental status and the aging process. We hope the information learned from this study will benefit the general public with aspects of physical activity and the aging process.

Privacy and Rights

Confidentiality is assured for all participants with regard to any responses and information you provide. All data collected will be numerically coded so that no individual data will be identifiable. If information from this study is published or presented at scientific meetings, your name and other personal information will not be used. Your personal information may be given out only if required by law. Organizations that may look at and/or copy your information and responses for research, quality assurance, and data analysis include:

- Government representatives, when required by law
- University of Illinois at Urbana-Champaign Institutional Review Board
- Primary care physician if the research staff, in the course of the project, learn of a medical condition that needs immediate attention

Participation in this project is voluntary and you are free to withdraw your participation without penalty at any time.

Costs

Participation in this aspect of the study is free. As an incentive, and appreciation for contributing your time to this study, you will be paid a stipend of $10 for each blood draw lab visit.

Contact Information

If you have questions at any time during your participation in this study, please feel free to contact Professor Arthur Kramer, Beckman Institute, 217/244-8373; Professor Edward McAuley, Department of Kinesiology and Community Health, 217/333-6487; Professor Jeffrey Woods, 217/244-8815; or Susan Herrel, Project Coordinator, 217/265-9848, toll free at 888/359-0022, or email: herrel@illinois.edu. Should you have further questions about your rights as a research participant, please contact the University of Illinois Institutional Review Board by email at irb@illinois.edu or by phone at 217/333-2670.

A description of this clinical trial will be available on: http://www.ClinicalTrials.gov, as required by U.S. Law and will be listed as "Influence of Fitness on Brain and Cognition II." This Web site will not include information that can identify you. At most, the Web site will include a summary of the results. You can search this Web site at any time.

I have read and understand the informed consent provided by Professors Edward McAuley, Arthur Kramer and Jeffrey Woods. I agree to the procedures outlines above and willingly consent to be a participant in this study. I have been given a copy of this consent form.

Name ____

Please print

Signature ____ ***Date*** __________

Name ______________________________
[Please print]

Fit & Active Seniors Trial (FAST)
at the University of Illinois at Urbana-Champaign
PARTICIPANT CONSENT FORM FOR BLOOD COLLECTION

Description

In this study we are asking you to donate a small amount of blood. We will be drawing a small blood sample and will give you instructions on how to prepare for this session in advance (e.g. overnight fasting). The maximal amount of blood drawn during any single sampling will be no more than 40mL (about 2.5 tablespoons), which is much less than that taken during one donation at the Red Cross Center or a blood drive (500 mL). The procedure will take approximately 10 minutes.

We will examine growth factors, inflammatory markers and oxidative stress levels in the blood. Specifically, these tests are:

- C-reactive protein [CRP] and Interleukin-6 [IL-6] which are proteins found in the blood that respond to inflammation levels in the body;
- Brain-derived neurotrophic factor (BDNF), a secreted protein that is related to brain function in the hippocampus, cortex and basal forebrain, areas related to learning, memory and higher thinking.

These measurements will be taken before you begin the FAST program and repeated six months later, at the end of the program. These blood samples will be stored frozen in the Exercise Immunology Research Laboratory in Freer Hall on the University of Illinois campus until the final analyses are complete. They will then be destroyed according to standard University protocol.

Formatted: Bulleted + Level: 1 + Aligned at: 0.25" + Indent at: 0.5"

Risks

The blood donation procedure is very common and involves minimal risk. There is a one in five chance of bruising in the area of sampling. This is generally not serious and will completely disappear within several days. As with all invasive procedures there is a slight risk of inflammation and infection. This risk will be minimized by the use of sterile procedures and equipment at all times. There is also a possibility of dizziness and lightheadedness associated with blood draws. However, you will be seated or lying down during and immediately following the blood draw, which will reduce the possibility of injury from a fall. These risks will also be minimized because a trained phlebotomist will draw all blood samples.

Benefits

There is no medical benefit to individuals who take part in this aspect of the study. We do, however, anticipate that participation in this project will contribute further to our understanding of the relation between physical, psychological, and mental status and the aging process. We hope the

information learned from this study will benefit the general public with aspects of physical activity and the aging process.

Privacy and Rights

Confidentiality is assured for all participants with regard to any responses and information you provide. All data collected will be numerically coded so that no individual data will be identifiable. If information from this study is published or presented at scientific meetings, your name and other personal information will not be used. Your personal information may be given out only if required by law. Organizations that may look at and/or copy your information and responses for research, quality assurance, and data analysis include:

- Government representatives, when required by law
- University of Illinois at Urbana-Champaign Institutional Review Board
- Primary care physician if the research staff, in the course of the project, learn of a medical condition that needs immediate attention

Participation in this project is voluntary and you are free to withdraw your participation without penalty at any time.

Costs

Participation in this aspect of the study is free. As an incentive, and appreciation for contributing your time to this study, you will be paid a stipend of $10 for each blood draw lab visit.

Contact Information

If you have questions at any time during your participation in this study, please feel free to contact Professor Arthur Kramer, Beckman Institute, 217/244-8373; Professor Edward McAuley, Department of Kinesiology and Community Health, 217/333-6487; Professor Jeffrey Woods, 217/244-8815; or Susan Herrel, Project Coordinator, 217/265-9848, toll free at 888/359-0022, or email: *herrel@illinois.edu*. Should you have further questions about your rights as a research participant, please contact the University of Illinois Institutional Review Board by email at *irb@illinois.edu* or by phone at 217/333-2670.

A description of this clinical trial will be available on: *http://www.ClinicalTrials.gov*, as required by U.S. Law and will be listed as "Influence of Fitness on Brain and Cognition II." This Web site will not include information that can identify you. At most, the Web site will include a summary of the results. You can search this Web site at any time.

I have read and understand the informed consent provided by Professors Edward McAuley, Arthur Kramer and Jeffrey Woods. I agree to the procedures outlines above and willingly consent to be a participant in this study. I have been given a copy of this consent form.

Name ____

Please print

Signature ____ ***Date*** __________

University of Illinois
at Urbana–Champaign

Institutional Review Board Office
528 East Green Street, Suite 203, MC-419
Champaign, IL 61820
tel: 217-333-2670
fax: 217-333-0405
E-mail: irb@illinois.edu
Web: www.irb.illinois.edu

IRB-1 Temp

Application for Review of Research Involving Human Subjects

IRB-1 v4 0510

This Section is for Office Use Only

UIUC IRB Protocol No. ______ Track: ______

Exempt under 45 CFR §46.101(b) ☐(1) ☐(2) ☐(3) ☐(4) ☐(5) ☐(6) Reviewer 1: ______

Expedite, Category ☐(1) ☐(2) ☐(3) ☐(4) ☐(5) ☐(6) ☐(7) ☐(8) ☐(9) Reviewer 2: ______

All forms must be completed, signed by the RPI, and submitted by FAX, Email, or single-sided hard copy.

Please, no staples!

1. RESPONSIBLE PROJECT INVESTIGATOR (RPI) The RPI must be a nonvisiting member of UIUC faculty or staff who will serve as project supervisor at UIUC. Students, interns, post-doctoral researchers, and visiting faculty from other campuses may not serve as RPI, but should be listed as Investigators, if applicable (see Part 3, below).

Last Name: Kramer	First Name: Arthur	Academic Degree(s): Ph.D.
Dept. or Unit: Beckman Institute	Office Address: 1st floor, Director's office	Mail Code: 251
Street Address: 405 N. Mathews	City: Urbana / State: IL	Zip Code: 61801
Phone: 217-244-8373	Fax:	E-mail: a-kramer@illinois.edu
UIUC Status: Nonvisiting member of (Mark One) ☒ Faculty ☐ Academic Professional/Staff		

2. PROJECT TITLE

Influence of Fitness on Brain and Cognition II

3. INVESTIGATORS List all investigators who are different from the RPI, including those from other institutions. Include all persons who will be directly responsible for the project's design or implementation, the consent process, data collection, data analysis, or follow-up. Collaborators, outside consultants, and graduate and undergraduate students should be listed if they will be responsible for these activities. Include all investigators named on grant proposals.

Last Name: McAuley	First Name: Edward	Academic Degree(s): Ph.D.
Dept. or Unit: Kinesiology and Community Health	Office Address: 336 Freer Hall	Mail Code: 052
Street Address: 906 S. Goodwin Ave.	City: Urbana / State: IL	Zip Code: 61801
Phone: 217-333-6487	Fax: 217-333-3124	E-mail: emcauley@illinois.edu
Affiliation:	UIUC ☒ Faculty ☐ Academic Professional/Staff ☐ Grad Student ☐ Undergrad Student ☐ Visiting Scholar, or ☐ Non-UIUC Affiliate of (Institution):	

Last Name: Woods	First Name: Jeffrey	Academic Degree(s): PhD
Dept. or Unit: Kinesiology and Community Health	Office Address: 348 Freer Hall	Mail Code: 052
Street Address: 906 S. Goodwin Ave.	City: Urbana / State: IL	Zip Code: 61801
Phone: (217) 244-8815	Fax: 217-333-3124	E-mail: woods1@illinois.edu
Affiliation:	UIUC ☒ Faculty ☐ Academic Professional/Staff ☐ Grad Student ☐ Undergrad Student ☐ Visiting Scholar, or ☐ Non-UIUC Affiliate of (Institution):	

List additional Investigators on an attachment and check here: ☒

4. RESEARCH STAFF. List other research personnel who should be copied on IRB Office correspondence for this study.

Last Name: Herrel	First Name: Susan	Academic Degree(s): M.S.
Dept. or Unit: Kinesiology and Community Health	Office Address: 334 Freer Hall	Mail Code: 052
Street Address: 906 S. Goodwin Ave.	City: Urbana	State: IL / Zip Code: 61801
Phone: 217-244-9848	Fax: 217-333-3124	E-mail: herrel@illinois.edu
Affiliation:	UIUC ☐ Faculty ☒ Academic Professional/Staff ☐ Grad Student ☐ Undergrad Student ☐ Visiting Scholar, or ☐ Non-UIUC Affiliate of (Institution):	

List additional Research Staff on an attachment and check here: ☒

5. FUNDING Indicate whether this research is funded by, or application has been made for, a grant, contract, or gift.

5A. STATUS
☐ Research is **not funded** and is **not pending** a funding decision (Proceed to Part 6).
☒ Research is **funded** (funding decision has been made).
☒ Funding decision is **pending**. Funding proposal submission date:

5B. SOURCE(S) If the research is funded or pending a funding decision, mark and name all sources:

	Type of Funding—check all that apply	Name of Source
☐	**UIUC Department, College, or Campus** (includes Research Board and Campus Fellowship Training Grants)	
☒	**Federal** (from federal agencies, offices, departments, centers)	National Institute on Aging
☒	**Commercial Sponsorship** (from corporations, partnerships, proprietorships)	Abbott Nutrition
☐	**State of Illinois Department or Agency** (from any state office or entity)	
☐	**Gift or Foundation (including UIF)** (public or private foundations, not-for-profit corporations, private gifts)	

→ Check here if the funding is through a Training Grant: ☐

5C. PROPOSAL Attach a complete copy of the funding proposal or contract. ☒ Attached

Sponsor-assigned grant number, if known: **R37 AG025667**

Title of Funding Proposal or Contract, if different from Project Title in Part 2:

N/A

5D. FUNDING AGENCY OFFICIAL, IF ANY, TO BE NOTIFIED OF IRB APPROVAL

Last Name: Wagster	First Name: Molly	Salutation: Dr.
Agency: NIA	Office Address: Gateway Bldg. Ste 350	Mail Code: 20814
Street Address: 7201 Wisconson Ave.	City: Bethesda	State: MD / Zip Code: 20892
Phone: 301-496-9350	Fax: 301-496-1494	E-mail: wagsterm@nia.nih.gov

6. FINANCIAL INTERESTS: Indicate below if any investigators or any members of their immediate families have any relationships, commitments, or activities with the sponsor of this research that might present or appear to present a conflict of interest with regard to the outcome of the research. (If a financial conflict of interest exists, please submit the UIUC approved conflict management plan. If you have questions about conflict of interest contact the Office of the Vice Chancellor for Research at 217-333-0034.)

☐ Ownership, equity or stock options
☐ Has been disclosed to the UIUC campus **OR** ☐ has not been disclosed to the UIUC campus

☐ Personal compensation such as royalties, consulting fees etc.
☐ Has been disclosed to the UIUC campus **OR** ☐ has not been disclosed to the UIUC campus
☐ Intellectual property such as patents, trademarks, copyright, licensing, etc.
☐ Has been disclosed to the UIUC campus **OR** ☐ has not been disclosed to the UIUC campus

☐ Other conflict of interest:

☐ Has been disclosed to the UIUC campus **OR** ☐ has not been disclosed to the UIUC campus

☒ No conflicts exist

7. SUMMARIZE THE RESEARCH. In **LAY LANGUAGE**, summarize the objectives and significance of the research.

See attachment.

If additional information summarizing the Research is attached, check here: ☐

8. PERFORMANCE SITES

Including UIUC sites, describe ALL the research sites for this protocol. For each non-UIUC site, describe: Whether the site has an IRB. Whether the site has granted permission for the research to be conducted. Contact information for the site. If the site has an IRB, whether the site's IRB has approved the research or planned to defer review to a UIUC IRB.		For non-UIUC sites, documentation of IRB approval is:
1.	Beckman Institute	☐ Attached ☐ Will Follow ☒ N/A
2.	Freer Hall	☐ Attached ☐ Will Follow ☒ N/A
3.	Illinois Simulator Laboratory	☐ Attached ☐ Will Follow ☒ N/A

List and describe any additional Performance Sites information on an attachment and check here: ☒

9. DESCRIBE THE HUMAN SUBJECTS

9A. SECONDARY DATA ONLY? If this research *only* involves the analysis of data that *has already been collected* from human subjects and *no new data collection will occur,* check here: ☐.

9B. MATERIALS OF HUMAN ORIGIN? Will this research involve the collection, analysis, or banking of human biological materials (*e.g.,* cells, tissues, fluids, DNA)? ☒ **Yes** ☐ **No** If yes attach **Appendix C**, the *Biological Materials Form.*

9C. ANTICIPATED NUMBERS How many subjects, including controls, will you study in order to get the data that you need? If you plan to study disproportionate numbers of a given sex, race, or minority group, provide scientific rationale in Part 11.

Performance Site		# Male	# Female	Total
1.	University of Illinois at Urbana-Champaign- Low active adults	125	125	250
2.	University of Illinois at Urbana-Champaign- ACTIVE adults	25	25	50
3.				
TOTALS		150	150	300

List Anticipated Numbers for additional Performance Sites on an attachment and check here: ☐

9D. AGE RANGE Mark all that apply. Researchers planning to include children in research projects involving *more than minimal risk* must provide written documentation of the benefits that are likely to accrue to a child participating in the project. This should include information gathered on adults, if it exists, or an explanation about why it does not exist.

☐ 0–7 years ☐ 8–17 years ☒ 18–64 years ☒ 65+ years

→ ☐ If applicable, written documentation of benefits for including children in ***more than minimal risk*** research is attached.

9E. SPECIAL OR VULNERABLE POPULATIONS Mark groups that will be targeted by design. Also indicate groups likely to be involved in the research even though they are not targeted by design.

☐ None of the following special populations will be targeted

☐ Children (age < 18 years)		
☐ Neonates	☐ Mentally disabled or cognitively impaired persons	
☐ Fetuses *(in utero)*	☐ Adults with legal guardians	
☐ *in vitro* fertilization subjects	☐ Persons with limited civil freedom (*e.g.,* members of military)	
☐ Pregnant or lactating women	☐ Specific racial or ethnic group(s)— describe:	
☐ Inpatients	☐ Low income or economically disadvantaged persons	
☐ Outpatients	☐ UIUC Students—name subject pool, if applicable:	
☒ Elderly (age > 65 years)	☐ Other College Students—name subject pool, if applicable:	
☐ Other (describe here):		

9F. If you checked any of the groups in question 9E, describe additional safeguards included in the protocol to protect the rights and welfare of special or vulnerable populations.

All individuals (staff and students) with direct contact with participants will maintain current CPR/AED and First Aid certification. In addition, prior to any exercise sessions, discussions will be held with local Fire Departments that will be first to respond to any adverse events that occur during research-related activities. These discussions will serve to inform the first responders about the days and times of exercise-related activities for this project and confirm protocols for emergency situations at each location. During exercise testing at Freer Hall, a physician and at least two fully trained graduate research assistants and several undergraduate research assistants will be on site to monitor the tests and the participants' safety. Included in the exercise testing room is a crash cart that is stocked with the necessary drugs and cardiac defibulator should the physician deem their use necessary. During exercise testing and exercise sessions, should any adverse events occur, the Exercise Psychology Lab has a set protocol in place that includes the contacting of emergency response personnel, facilitating their arrival, contacting the participant's personal physician with information regarding the event, and follow-up. With regard to the fMRI testing of older adults' cognitive and brain function, measures have been taken to ensure participant safety. All guidelines set forth by the Society for Psychophysiological Research will be followed during data collection. Further, all MRI equipment has received FDA approval for safety. For blood sampling, standard antecubital venipuncture will be performed by a trained phlebotimist using sterile technique. Finally, issues related to participant safety during the exercise intervention trials, exercise stress testing, and cardiovascular monitoring will be discussed on a weekly basis as a fixed agenda item at our laboratory meetings to ensure constant monitoring of our safety procedures.

If additional Item 9E information is attached, check here: ☐

10. RECRUITMENT

10A. RECRUITING PROCEDURES Specifically describe the systematic procedures for finding and recruiting subjects or requesting pre-existing data or materials. How will voluntary participation be ensured? State whether any of the researchers are associated with the subjects (*e.g.,* subjects are students, employees, patients). Name any specific agencies or institutions that will provide access. Who will contact the prospective subjects? Who gives approval if subjects are chosen from records? Describe solicitation through the use of advertising (*e.g.,* posters, flyers, announcements, newspaper, radio, television, Internet), face-to-face interaction, direct mail or phone contact, classrooms, subject pools, health care registries, patient referrals, and institutional "gatekeepers," as applicable.

Low active older adults will be drawn from the Urbana-Champaign and Champaign County area and will be recruited through a variety of media that will advertise the opportunity to participate in a research project that involves a free six-month physical activity program. A variety of advertising outlets and methods will be used for this project including but not necessarily limited to: print media (newspapers, magazines, newsletters); flyers; email, social network, Websites and listserve announcements; television and radio; university, organizational and departmental database lists; presentations; and public service announcements.
Advertising for these low active adults will include the following information as time and/or space permits: Healthy adults between the ages of 60-79 years of age are needed for a research project examining the effects of physical activity on health and well-being. Participants will be involved in a free, supervised six-month physical activity program that will take place on the UI campus. Parking will be provided at no cost. To be considered for participation, adults must: be between 60-79 years of age; not be currently exercising on a regular basis any more than two days/week; be able to walk on a treadmill; be able to provide physician's release to exercise; live independently; be right handed; have correctable vision; be able to submit to an MRI of the brain; have no history of stroke; be English-speaking; be able to come to the UI campus for regular physical activity sessions during the six-month study period.
Potential participants who respond to advertising will be instructed to call or email the research coordinator in charge of this trial. She or trained research specialists will respond to the contact and conduct the first screening interview by telephone. If participants respond by email, a follow-up email will be sent (see attached) with a request for contact information. The pre-screening telephone script is also attached.
In addition, we will recruit 50 HIGH ACTIVE adults who will participate in all baseline assessments only. These individuals will be recruited separately with flyers and emails to master athlete organizations such as Master Swimming Club, Second Wind Running Club, Tennis Clubs etc. We may utilize all of the advertising outlets listed above but will emphasize the activity level component.
Advertising for the ACTIVE adults will include the following information as time and/or space permits: Healthy and active adults between the ages of 60-79 years of age are needed for a research project examining the effects of physical activity on health and well-being. Parking will be provided at no cost. To be considered for participation, adults must: be between 60-79 years of age; currently exercising on a regular basis at least four days/week; be able to walk on a treadmill; be able to provide physician's release to exercise; live independently; be right handed; have correctable vision; be able to submit to an MRI of the brain; have no history of stroke; be English-speaking. A telephone screening script and an email response are attached and labeled as "ACTIVE Adults".

If additional Item 10A information is attached, check here: ☒

Attach final copies of recruiting materials including the final copy of printed advertisements and the final version of any audio/taped taped advertisements and check here: Attached ☐ Will Follow ☒

10B. WITHHELD INFORMATION Do you propose to withhold information from subjects prior to or during their participation?
☐ Yes ☒ No

If yes, describe what will be withheld, justify the withholding (address risks, provide rationale), describe the debriefing plan, and attach a labeled copy of a written debriefing form, to be provided to subjects. ☐ Debriefing Attached ☐ Will Follow

If additional Item 10B information is attached, check here: ☐

10C. PROTECTED HEALTH INFORMATION (PHI) The IRB must address the privacy and use of health information that is created, received, or housed by health care providers, health plans, or health care clearinghouses and that identifies or could be used to identify an individual. During *either recruiting or data collection*, will you use or have access to such information that is related to the past, present or future health or conditions of a *living or deceased* individual, provision of health care to the individual, or the payment for the provision of health care to the individual? ☐ Yes ☒ No

10D. SCHOOLS-BASED RESEARCH If subjects will be recruited from Illinois public or private elementary or secondary schools, additional deadlines and procedures apply. Criminal background clearances might be required. Special consideration must be given to the exclusion of protected populations. Please contact the Office of School–University Research Relations (OSURR) (217.244.0515 or http://www.ed.uiuc.edu/BER/OSURR.html) for more information. Mark one:

☐ Illinois schools **will** be used ☒ Illinois schools **will not** be used

11. INCLUSION AND EXCLUSION CRITERIA Address all four of the following items in explaining who will and will not qualify for participation and how that determination will be made: (1) Describe procedures to assure equitable selection of

subjects. Justify the use of any special or vulnerable groups marked in Part 9E. Selection criteria that target one sex, race, or ethnic group require a clear scientific rationale. (2) List specific criteria for inclusion and exclusion of subjects in the study, including treatment groups and controls. (3) Name and attach copies of measures and protocols that will be used to screen applicants. (4) Explain how the inclusion/exclusion criteria will be assessed and by whom. If special expertise is required to evaluate screening responses or data, tell who will make this evaluation and describe their training and experience.

Two tables of inclusion and exclusion criteria are attached. One for the Low Active adults and one for the ACTIVE adults.

If additional Item 11 information is attached, check here: ☒

12. RESEARCH PROCEDURES: Using <u>LAYMAN'S LANGUAGE</u>, specifically describe what the participants (treatment groups and controls) will do and where the research activities will take place. Give approximate dates and durations for specific activities, including the total number of treatments, visits, or meetings required and the total time commitment. (For schools-based research where class time is used, describe in detail the activities planned for nonparticipants and explain where (*e.g.,* in a classroom, in a private area) both participants and nonparticipants will be located during the research activities. Include a concise description of procedures, locations, time commitments, and alternate activities on the relevant consent and assent forms.)

See separate additions document.

If additional Procedures are attached, check here: ☒

13. EQUIPMENT Will any physical stimulation or physiological data acquisition equipment be used with the subjects?
☒ Yes ☐ No If yes, attach **Appendix A**, the *Research Equipment Form*.

14. DRUGS, DEVICES, AND BIOLOGICS Will any drugs, devices, or chemical or biological agents be used with the subjects?

☐ Yes ☒ No If yes, attach **Appendix B**, the *Drugs, Agents, and Devices Form*.

15. MRI AT BIC To use the Beckman Institute Biomedical Imaging Center (BIC) in human subject's research, you must obtain *prior approval* from the BIC (217.244.0600; bmrf@bmrl.bmrf.uiuc.edu) and use BIC-approved screening and consent forms. Attach:

BIC approval	☒ Attached
BIC screening form	☒ Attached
BIC consent form	☒ Attached

16. MEASURES If subjects will complete questionnaires, surveys, interviews, psychological measures, or other measures, however administered, the IRB must review and approve the measures. List all such measures here and attach complete, labeled copies (including translations, if applicable) to this application:

Measure 1:	See attached list for comprehensive list of measures and administration schedule for both the Low active and ACTIVE adults	☒ Attached	☐ Will Follow
Measure 2:		☐ Attached	☐ Will Follow
Measure 3:		☐ Attached	☐ Will Follow
Measure 4:		☐ Attached	☐ Will Follow

List additional Measures on an attachment and check here: ☒

17. SUBJECT REMUNERATION

Will subjects receive inducements or rewards before, during, or after participation? ☒ Yes ☐ No
If yes, will payment be prorated for partial participation? ☒ Yes ☐ No
If remuneration will be given, for each subject group:

(1) specify the form of remuneration, including $, course credit, lottery, gift certificate, or other;
(2) state the $ amount or the approximate $US value, or the course credit and its percentage of the final grade;
(3) explain the remuneration plan, including whether and how prorating will be made for partial participation;
(4) for lotteries, include (a) the number of prizes, (b) the nature and value of each prize, (c) the approximate odds of winning, (d) the date(s) of the drawing(s), and (e) how winners will be notified, by whom, and by when; and
(5) include all this information on the relevant consent forms.

Participants who are low active will be paid for the testing sessions completed in the following manner:

- $10 for Mock MRI appointment 1hr. appointment (x1) = $10 (baseline only)
  $10 for Blood Draw appointment 10 minute appointment (x2) = $20 (baseline, m6)
- $10 per hour for the Neuropsychological appointments.(x2) 2hr. = $40 (baseline, m6)
- $10 per hour for street crossing appointment 2hr. appointment (x2) = $40 (baseline, m6)
- $20 per hour for the fMRI appointment. 2hr. appointment (x2) = $80 (baseline, m6)
- $20 per test for the Treadmill test. 1hr. appointment (x2) = $40 (baseline, m6)

All renumeration will be prorated by test. Total paid if all testing completed=$230.

Participants who are high ACTIVE will be paid for the testing sessions completed in the following manner:

- $10 for Mock MRI appointment 1hr. appointment = $10
- $10 per hour for the Neuropsychological appointments. 2hr. = $20
- $10 for street crossing appointment 2hr. appointment = $20
- $20 per hour for the fMRI appointment. 2hr. appointment = $40
- $20 per test for the Treadmill test. 1hr. appointment = $20

All renumeration will be prorated by test.

If additional Subject Remuneration information is attached, check here: ☐

18. SUBJECT OUTLAY Will subjects incur costs for research-related procedures (*e.g.*, longer hospitalization, extra tests), use of equipment, lost compensation, or transportation (over 50 miles)? ☐ Yes ☒ No If yes, describe here:

If additional Subject Outlay information is attached, check here: ☐

19. CONFIDENTIALITY OF DATA Answer each of the following to describe methods that will ensure the confidentiality of individually identifiable data. Confidentiality is required unless subjects give express, written permission to have their identifiable information published, presented, or shared.

19A. CHECK IF USED IN DATA COLLECTION: ☐ Audio tapes/ Digital voice ☐ Video tapes ☐ Still photos ☒ Other imaging

19B. DATA COLLECTION Explain how the data will be collected. If anonymous data collection is proposed, provide details of how investigators *will not have the ability to trace responses to subject identities.* For multiphase data collection or if multiple contacts will be made with subjects, specifically explain the subject tracking and coding systems.

Address the confidentiality of data collected via e-mail, databases, Web interfaces, computer servers, and other networked information, as applicable.

Data will be coded by study ID number provided for each participant. Only research personnel will be able to identify a participant by his/her study ID number unless otherwise requested by state or federal law.
Data will be checked immediately in the presence of the research participant if possible for clarity and completeness at time of collection. For data provided by mail, follow-up telephone calls to clarify missing data will be made. If necessary, incomplete or ambiguous results will be corrected with follow-up telephone calls and/or additional appointments with research staff.
Data will be coded and double entered directly on a computer by experienced data entry personnel. Once command files have been created, frequency distributions for the measures will be examined to check for missing information and out-of-range values. System files will then be created once all errors in the raw data have been corrected.
All investigators are connected via a secure network, which is accessible only by password and therefore provides restricted access to the data.
To ensure the integrity of the data collected from study participants, several procedures will be implemented. All personnel involved in data collection will be thoroughly trained in assessment methods thus ensuring consistent applications of procedures and measurement consistency across participants. All data will be automatically saved to a computer hard drive in real time and then stored on a secure server (i.e. password protected) which is backed up daily. All raw data will be transformed to PDF files and saved on two external hard drives, which will be stored in a locked cabinet in Professor McAuley's laboratory in Freer Hall. Medical histories will be maintained as hard copies, however, for referral during exercise testing. Original copies of Informed Consents will be kept in dedicated locked cabinets, separate from other participant files. Study investigators will be responsible for overseeing data collecion, entry and management. All aspects of data quality control and analysis will be supervised by the PIs.
Continuing review of all procedures will be obtained by the University of Illinois Institutional Review Board according to UI policies and procedures.

If additional Item 19B information is attached, check here: ☐

19C. DATA SECURITY Describe how and where the data be kept so that the data remain confidential.

All raw data will be de-identified (with the exception of the study ID number). All informed consents and medical histories will be kept in separate locked file cabinets with restricted access. Issues related to data integrity will be discussed on a weekly basis as a recurring agenda item in the weekly laboratory meetings.The data are backed up on secure external hard drives. After data have been entered and checked, PDFs will be made of the original hard copies and these documents will be shredded with confidential University documents. PDFs will be stored in study specific external hard drives, which will be stored in a locked and secure cabinet with restricted access.

If additional Item 19C information is attached, check here: ☐

19D. STAFF TRAINING Describe the training and experience of all persons who will collect or have access to the data.

Only the investigators will have access to participant data. All staff with access to the data will have completed the UIUC IRB Web-based tutorial in the responsible conduct of Research and the Collaborative Institutional Training Initiative (CITI) Training Modules. The graduate students and post-docs who will be collecting data are well versed in research ethics and confidentiality since this information is conveyed in Professor Kramer and McAuley's lab meetings and is also received in formal training in research ethics and principles in both the Department of Psychology and the Department of Kinesiology. The students participating in different facets of the project (exercise testing, exercise training, MRI recording) have been trained by Professors Kramer and McAuley in the appropriate methods and procedures. All research assistants who have contact with participants will have current CPR, AED and First Aid certifications.

If additional Item 19D information is attached, check here: ☐

19E. DATA RETENTION How long will the data be kept?

The data will be kept for 5 years after publication as per requirements of the American Psychological Association.

If additional Item 19E information is attached, check here: ☐

19F. DISSEMINATION OF RESULTS What is(are) the proposed form(s) of dissemination (*e.g.*, journal article, thesis or academic paper, conference presentation, sharing within industry or profession)?

Journal articles, conference presentations, book chapters, and graduate student theses.

If additional Item 19F information is attached, check here: ☐

19G. PRIVACY Describe provisions to protect the privacy interests of subjects.

Each participant will be assigned a random unique identification (ID) number as the only way to match data to a specific person. An electronic database stored on a secured, password protected network only accessible by the study investigaors will house participant contact information and ID number. All written hard copies of questionnaire data will only have the ID number written on them and will be stored securely as described above. The key will be destroyed upon study completion.

If additional Item 19G information is attached, check here: ☐

19H. INDIVIDUALLY IDENTIFIABLE INFORMATION Will any individually identifiable information, including images of subjects, be published, shared, or otherwise disseminated? ☒ Yes ☐ No

If yes, subjects must provide explicit consent or assent for such dissemination. Provide appropriate options on the relevant consent documents.

20. INFORMED CONSENT: University policy requires the execution of a comprehensive, written document that is signed by the subject (or the subject's authorized representative) as the principal method for obtaining consent from subjects.

The language in the document must be understandable to the subject or the subject's legally authorized representative. A Waiver or Alteration of Informed Consent or a Waiver of Documentation (signature) of Informed Consent (e.g., online consent, oral consent) may be approved by the IRB. If requesting a waiver please complete the appropriate waiver form at: www.irb.illinois.edu and submit it with the IRB Application for review.

Children must *assent* (or, voluntarily agree) to participation and a parent must separately consent on behalf of their child (*i.e.*, two different forms are generally required). Children under age 8 may assent either orally or passively, depending on their level of maturity. Children 8–17 years old should sign a written form unless the UIUC IRB approves a different process.

20A. TYPE OF CONSENT Check all that apply and attach one copy of each relevant form, letter, or script on university letterhead. Include translations, if consent will be obtained in a foreign language. Use headings, headers, or footers to uniquely identify each document and associate it with the subject group for which it will be used.

☒ **Written informed consent (assent) with a document <u>signed by</u>**

☒ adult subjects ☐ parent(s) or guardian(s) ☐ adolescents aged 8–17 years

☒ **Waiver or Alteration of Informed Consent (Attach waiver form.)**
☒ adult subjects ☐ parent(s) or guardian(s) ☐ adolescents aged 8–17 years

☐ **Waiver of Documentation (signature) of Informed Consent (Attach waiver form.)**
☐ adult subjects ☐ parent(s) or guardian(s) ☐ adolescents aged 8–17 years

20B. USE OF PROXY Will others (*e.g.,* next of kin, legal guardians, powers of attorney) act on behalf of adult subjects in giving consent to participate in this research? ☐ Yes ☒ No if yes, describe in Section 20D.

20C. USE OF PROXY OUTSIDE THE UNITED STATES If a proxy is used in research conducted outside Illinois, provide justification (e.g., statement of an attorney or copy of applicable law) that the proxy is authorized under the laws of the jurisdiction in which the research will be conducted to consent to the procedures involved in this protocol.

20D. CONSENT PROCESS Describe when and where voluntary consent will be obtained, how often, by whom, and from whom. If cognitively impaired subjects (including children under age 8) will be involved, explain how the subject's understanding will be assessed and how often; include the questions that will be asked or actions that will be taken to assess understanding.

Describe any waiting period between informing the prospective subject and obtaining the consent. Describe steps taken to minimize the possibility of coercion or undue influence. Indicate the language used by those obtaining consent. Indicate the language understood by the prospective subject or the legally authorized representative.

If the research involves pregnant women, fetuses, or neonates, indicate whether consent will be obtained from the mother, father, or both. If the research involves children, indicate whether consent will be obtained from: Both parents unless one parent is deceased, unknown, incompetent, or not reasonably available, or when only one parent has legal responsibility for the care and custody of the child; or from one parent regardless of the status of the other parent.

After it has been determined that potential participants meet the study inclusion criteria, they will be extended an offer to participate in the study. No participation in any part of the testing or intervention will take place until potential participants have read through and signed a paper copy of an informed consent written at a level that should be understood by all adults. All participants will be provided with a copy of the informed consent. The informed consent will clearly state that participation is voluntary and may be discontinued at any time without penalty. Participants will be encouraged to ask any questions they may have pertaining to the informed consent or participation in the intervention.
During the initial screening phone call, information regarding their physical activity and medical history will be discussed with the participant to determine eligibility. We have requested a waiver of informed consent as we will retain basic demographic data of those participants who do not pass screening to conform with standard reporting criteria for clinical trials.
A standard EPL photo release will be presented to participants prior to participation in the intervention programs. A record will be kept of those who choose not to sign the release and the document will be stored in the participant's file.

If additional Item 20D information is attached, check here: ☐

21. RISKS

21A. DESCRIPTION Specifically describe all known risks to the subjects for the activities proposed and describe the steps

that will be taken to minimize the risks. Include any risks to the subject's physical well-being, privacy, dignity, self-respect, psyche, emotions, reputation, employability, and criminal and legal status. Risks must be described on consent forms.

Risks of exercise testing and exercise participation: As indicated on the consent form participants are informed that individuals who have been sedentary have a chance of incurring minor injury and some discomfort due to intensified use of major muscle groups that have not received a great deal of use. However, no major injuries are anticipated given that exercise leaders employ best practices in fitness training. Participants are encouraged to notify their exercise leaders and physicians if they become injured while participating in the trial. There is also a very slim chance that sudden death or cardiac irregularities can occur while exercising. This is very rare and the benefits of exercise are known to outweigh the risks. As a preventive measure, during all on-site physical assessments, all staff members are First Aid and CPR certified.
Risks of MRI session: MRI scanning can be hazardous in the presence of metallic devices. Hence participants are carefully screened for metal implants (see MRI consent form). Although highly unlikely, individuals may experience dizziness, nausea, headache, flashing lights, unusual tastes, numbness, or tingling while in the magnet, or possible momentary loss of balance while leaving the magnet. These sensations are mostly due to movement while inside the magnet and can be minimized by holding still. All of these sensations stop shortly after an individual leaves the magnet. We help participants get up when they leave the magnet to ensure that they do not lose their balance. Some individuals also experience claustrophobia in tight spaces and this can be experienced in the magnet. Therefore, we screen for claustrophobia prior to an individual entering the magnet and also ensure that they can leave the magnet at any time if they feel uncomfortable.
Risks for blood collection:
The blood donation procedure is very common and involves minimal risk. There is a one in five chance of bruising in the area of sampling. This is generally not serious and will completely disappear within several days. As with all invasive procedures there is a slight risk of inflammation and infection. This risk will be minimized by the use of sterile procedures and equipment at all times. Risk will also be minimized because a trained phlebotomist will draw all blood samples.

If additional Risks information is attached, check here: ☒

21B. RISK LEVEL: ☐ **No more than minimal risk**
(the probability and magnitude of harm or discomfort anticipated for participation in the proposed research are not greater in and of themselves than those ordinarily encountered in daily life or during the performance of routine physical or psychological examinations or tests).

☒ **More than minimal risk**

21C. Data Monitoring Plan: If you checked that the research is more than minimal risk, describe the provisions for monitoring the data to ensure the safety of subjects (Who will periodically monitor harms and benefits experienced by subjects to ensure that the relationship of risks to potential benefits remains unchanged? How often will monitoring occur? What analyses will be performed? If appropriate, what criteria will be used to stop the research based on monitoring of the results?)

All study data and adverse events will be monitored and evaluated by the PIs on a regular, weekly basis. A data safety and monitoring board (DSMB) has been established to ensure the safety of all participants and the validity and integrity of the data collected. The board includes a physican and two external academics with expertise in physical activity interventions with older adults. These members will be independent of the study investigators. The DSMB will meet at 6-month intervals to evaluate all study protocols, the ongoing safety of participants, and the integrity of the data. The meetings will be via telephone conferencing. Adverse events and serious adverse events will be reported using the UIUC IRB forms and will follow standard protocol.

22. BENEFITS Describe the expected benefits of the research to the subjects and/or to society.

Participants who take part in the physical activity intervention may experience improved physical, mental, and emotional well-being, enhanced endurance and improved quality of life. However, no promise of a direct benefit will be made to the participants. Results from the study may be beneficial to other sedentary older adults. It is important to confirm the most effective physical activity behavior change intervention components for translation of the intervention to a broader range of clinical and community settings. Moreover, understanding the mechanisms of physical activity behavior will enhance future intervention design and effectiveness. Such information may potentially lead to improved health, better quality of life, and reduced fatigue in older adults.Given the poor physical condition of many older adults, the high levels of physical inactivity in this population, and the existing body of knowledge supporting the beneficial effects of physical activity in older adults, we believe the benefits of participating in this study substantially outweigh the minimal risks described above.

Participants who take part in the ACTIVE adult study may experience improved physical, mental, and emotional well-being, enhanced endurance and improved quality of life from participation in physical activity. However, no promise of a direct benefit will be made to the participants. Results from the study may be beneficial to other active older adults. It is important to confirm the most effective physical activity behavior components for translation of the findings to a broader range of

clinical and community settings. Moreover, understanding the mechanisms of physical activity behavior will enhance future intervention design and effectiveness. Such information may potentially lead to improved health, better quality of life, and reduced fatigue in older adults. Given the poor physical condition of many older adults, the high levels of physical inactivity in this population, and the existing body of knowledge supporting the beneficial effects of physical activity in older adults, we believe the benefits of participating in this study substantially outweigh the minimal risks described above.

If additional Benefits information is attached, check here: ☐

23. RISK/BENEFIT ASSESSMENT Weigh the risks with regard to the benefits. Provide evidence that benefits outweigh risks.

Given the poor physical condition of many older adults, the high levels of physical inactivity in this population, and the existing body of knowledge supporting the beneficial effects of physical activity in older adults, we believe the benefits of participating in this study substantially outweigh the minimal risks described above.

If additional Risk/Benefit information is attached, check here: ☐

24. Is this a multi-center study in which the UIUC investigator is the lead investigator of a multicenter study, or the UIUC is the lead site in a multi-center study. **Yes** ☐ **No** ☒

If yes, describe the management and communication of information obtained that might be relevant to the protection of subjects, such as: Unanticipated problems involving risks to subjects or others. Interim results. Protocol modifications.

25. INVESTIGATOR ASSURANCES: The original signature of the Responsible Project Investigator is required before this application can be processed (scanned or faxed signatures are acceptable). Other investigators are also responsible for these assurances and are encouraged to sign. Neither stamps nor proxy signatures are accepted in this section.

I certify that the information provided in this application, and in all attachments, is complete and correct.

I understand that I have ultimate responsibility for the protection of the rights and welfare of human subjects, the conduct of this study, and the ethical performance of this project.

I agree to comply with all UIUC policies and procedures, the terms of its Federal Wide Assurance, and all applicable federal, state, and local laws regarding the protection of human subjects in research.

I certify that

- the project will be performed by qualified personnel according to the UIUC IRB-approved protocol.
- the equipment, facilities, and procedures to be used in this research meet recognized standards for safety.
- no change will be made to the human subjects protocol or consent form(s) until approved by the UIUC IRB.
- legally effective informed consent or assent will be obtained from human subjects as required.
- Unanticipated problems, adverse events, and new information that may affect the risk–benefit assessment for this research will be reported to the UIUC IRB Office (217.333.2670; irb@illinois.edu) and to my Departmental Executive Officer.
- I am familiar with the latest edition of the UIUC *Handbook for Investigators*, available at www.irb.illinois.edu, and I will adhere to the policies and procedures explained therein.
- student and guest investigators on this project are knowledgeable about the regulations and policies governing this research.
- I agree to meet with the investigator(s), if different from myself, on a regular basis to monitor study progress.
- if I will be unavailable, as when on sabbatical or other leave, including vacation, I will arrange for an alternate faculty sponsor to assume responsibility during my absence. I will advise the UIUC IRB by letter of such arrangements.

I further certify that the proposed research has not yet been done, is not currently underway, and will not begin until IRB approval has been obtained.

NOTE: The original signature of the RPI must be submitted before IRB Review (scanned or faxed signatures are acceptable).

Responsible Principal Investigator	Date	Investigator	Date
[signature]	3/21/12		
Investigator	Date	Investigator	Date

25. **(OPTIONAL) DEPARTMENTAL ASSURANCE** To be completed by the RPI's Departmental Executive Officer or their designee (proxy and stamped signatures are acceptable).

The activity described herein is in conformity with the standards set by our department and I assure that the principal investigator has met all departmental requirements for review and approval of this research.

Departmental Executive Officer (or designee) Date

IRB-1 additions

3. Investigators

Fahey, George C., PhD. Department of Animal Sciences, 166 ASL, 127 W. Gregory Dr., Urbana, IL 61801, MC-630, 217-333-2361, gcfahey@illinois.edu UIUC Faculty

Cohen, Neal J. PhD. Beckman Institute, 1624 Beckman Institute, 405 N. Mathews, Urbana, IL 61801, MC-251, 217-244-4229, njc@illinois.edu

Voss, Michelle. PhD. Department of Psychology, E11 Seashore Hall, Iowa City, IA 52242-1409, 319-335-2450, michelle-voss@uiowa.edu

4. Research staff

Erin Olson, 906 S. Goodwin Ave. Freer Hall Room 340, Urbana, IL 61801 , 217-244-4510

Amanda Szabo, 906 S. Goodwin Ave. Freer Hall Room 338, Urbana, IL 61801, 217-333-2427

Thomas Wójcicki, 906 S. Goodwin Ave. Freer Hall Room 338, Urbana, IL 61801, 217-333-2427

Neha Gothe, 906 S. Goodwin Ave. Freer Hall Room 338, Urbana, IL 61801, 217-333-2427

Emily Mailey, 906 S. Goodwin Ave. Freer Hall Room 340, Urbana, IL 61801 , 217-244-4510

Jason Fanning, 906 S. Goodwin Ave. Freer Hall Room 338, Urbana, IL 61801, 217-333-2427

Sean Mullen, 906 S. Goodwin Ave. Freer Hall Room 332, Urbana, IL 61801 , 217-244-4502

Laura Chaddock, Beckman Institute, Urbana, Illinois, 217- 244-1933

Chelsea Wong, Beckman Institute, Urbana, Illinois, 217- 244-1933

Ruth Franklin Sosnoff, 906 S. Goodwin Ave. Freer Hall Room 334, Urbana, IL 61801, 217-333-3180

James Monti, 405 N. Matthews Ave Beckman Institute 2424, Urbana, IL (217)244-1618

7. Summarize the research

We propose to test the hypothesis that a six-month intervention of combined fitness and cognitive training in the form of dancing will have a significant positive effect on a variety of executive control and memory processes as well as brain structure and function as compared to a non-aerobic strength and balance control/comparison group.

Our previous trial (known as HALT, Healthy Active Lifestyle Trial, IRB #94124) as well as that of others suggests that improvements in aerobic fitness have beneficial effects on cognitive function that are rather specific. That is, improvements in aerobic fitness appear to result in improvements in executive control processes such as scheduling, planning, coordination, inhibition, and working memory – some of the very cognitive abilities most affected during aging. Indeed, executive control processes and the prefrontal and frontal regions which support them have shown substantial and disproportionate age-related declines.

The main hypothesis that we test in the present project is that such deficits may be particularly benefited by improvements in aerobic fitness combined with cognitive training in the form of dance. Furthermore, we are hypothesizing that a nutritional supplement combined with a more traditional form of aerobic training i.e. walking will show equal or additional cognitive benefits to that of the aerobic/cognitive training exercise. We will investigate the relationship between aerobic fitness, physical activity, cognitive status, and brain function with this six-month aerobic training intervention study. We will collect psychosocial data, functional fitness data, and analyze protein in the blood to assess other relevant changes in psychological and physical function. Healthy, non-active older adults (60 to 79 years of age) will be recruited from the local community. The participants will be randomly assigned to one of four groups: an aerobic/cognitive combination group (dance); a moderate intensity walking group; a moderate intensity walking group who also are given a nutritional supplement; a non-aerobic control group (stretching, strengthening, and stability). The exercise interventions will be conducted by trained exercise staff. The participants will be assessed before and after the intervention (6 months). Assessments will include (a) cardiorespiratory testing, (b) physical activity monitoring (c) performance on neurocognitive tests of executive and non-executive function (d) measures of brain activation (fMRI) during cognitive tasks in a 3.0 tesla MRI system (e) a battery of psychosocial questionnaires (f) functional performance measures and (g) a mock street walking task (h) and blood sampling.

8. Performance Sites

Multipurpose room and walking track at Activities Recreation Center ARC) and Campus Recreation Center East (CRCE)

Beckman Institute, LifeLong Learning Laboratory

Beckman Institute, Biomedical Imaging Center

10A. Recruitment procedures

Initial phone call prescreening protocol

INTRODUCTION:

Hello. This is ________________ with the University of Illinois, Department of Kinesiology and Community Health. I'm returning your call about the Physical Activity Program. [*exchange basic pleasantries – How are you today?*]

I would like to tell you about the program and if you are still interested, I will need to ask you some questions to make sure that you are eligible. Do you have time to talk right now?

a. **NO**—When would be a good time for me to give you a call back? – *indicate in DB when to call back.*

b. **YES**—Ok, great. [continue to contact information and demographics]

If they are not home leave the following message: Hello. This is ________________ with the University of Illinois, Department of Kinesiology and Community Health. I'm returning your call about the research project involving a physical activity program. Please feel free to call us again at 217/265-9848. Thank you.

CONTACT INFORMATION AND DEMOGRAPHICS:

Before we go too much further, may I have your name and some basic contact information?

– complete in DB under Contact information tab.

I would also like to get some basic demographic and general health information. This information is kept in a secure database in our lab and if there are any questions that you prefer not to answer, you don't have to. This information will help us verify that our advertising has reached a diverse representation of our area.

– complete in DB under Screening tab. [Continue through the questions about health and then to the exclusion questions.]

Thank you. Now let me tell you about the program.

DESCRIPTION OF PROGRAM:

The National Institute on Aging and the Center for Nutrition, Learning and Memory at the UI have funded this study to determine the effects of physical activity training on how you process your thoughts and how your brain functions. The study is known as Fit & Active Seniors Trial (FAST) at the University of Illinois at Urbana-Champaign. We are looking for adults between 60 – 79 years of age who do not regularly exercise to participate in a free six-month physical activity program. You would be *randomly assigned* to one of four groups:

- A group that focuses on stretching, strengthening, and stability work;
- A group that focuses on dancing such as English Country or Contra Dancing ;
- A group that focuses on aerobic training in the form of walking; or
- A group that focuses on aerobic training in the form of walking while also taking a nutritional supplement that has been developed specifically for this study.

The groups are supervised by trained exercise and nutritional specialists. Again, I would like to mention that the group placement is entirely random and I cannot guarantee which group you will be in.

All groups will meet three times a week for six months [insert date or approximate] – Mondays, Wednesdays, and Fridays at 6:45 a.m. at different locations on the UI campus. Parking will be provided at no cost to you.

Everyone must be able to provide their physician's consent for them to be involved in the testing and be a part of the exercise program. We will facilitate this process.

As a requirement of the study, we will be asking you to perform some **psychological and fitness tests that include:**

- **A blood sampling.** A trained phlebotomist will draw approximately 2T of blood for inflammatory and protein analysis.
- **A series of neuropsychological tests.** These are both computer-based and paper and pencil tests that will examine different aspects of your attention, memory and decision-making. For example, you may be presented with a folded piece of paper and be asked to make the shape from sight. You will also be asked to remember things like shapes and numbers that you have seen earlier. These take place at the Beckman Institute on the UI campus.
- **A virtual street crossing.** You will be asked to walk on a self-propelled treadmill while crossing a virtual street that will be presented on a screen in front of you. You will also be asked to do this with the distraction of talking on a cell phone. This test will take place at the Beckman Institute Illinois Simulator Lab on the south side of the UI campus.
- **A functional MRI of the brain.** For this test, you will perform a series of question and answer tasks while we are collecting images of your brain at work. The amount of time

you will spend in the MRI machine will be 90 minutes. We will ask you to attend a "Mock MRI" prior to this to make sure that you will be able to tolerate the actual MRI in terms of comfort and claustrophobic issues. Although this is not a full-body MRI and these are scans of your brain only, you are placed in the magnet to approximately your waist and we will need to evaluate your ability to complete this test.

- **A graded exercise test.** This is a walking test on a treadmill. We will ask you to walk at a comfortable speed and we will adjust the grade (or the incline) of the treadmill to make it more challenging. You will wear a mask over your mouth and nose so that we can measure the air you use while exercising. You will walk until you feel you cannot walk any more.

All of these tests will take place on the UI campus. There are also a few things that we will ask you to do at home:

- We will ask you to wear an **accelerometer** for seven days. This is a device very much like a pedometer (but it records all activity not just your steps); you wear it on a strap around your waist.
- We will also ask you **complete a series of paper-and-pencil questionnaires** that you can do at home.

You will repeat these assessments at the end of the program (6 months later).

No matter which group you are assigned to, you will be paid in the following manner for completing these assessments that I just mentioned:

- Neuropsychological appointments – 2 hours of your time. Total $20 (2 times)
- Blood draw -- ½ hour of your time. Total $10 (2 times)
- Street crossing – 2 hours of your time. Total $20 (2 times)
- fMRI – 2 hours of your time. Total $40 (2 times)
- Treadmill test – 1 hour. Total $20 (2 times)

You also receive $10 for the Mock MRI. Thus, you can expect to receive $230 for being a part of this study if you complete all the assessments. And another reminder, there will be no cost to you for parking when you come for testing and the exercise sessions.

The benefits for you will be the free physical activity program as well as making a contribution to the science and understanding of physical activity and the aging process.

Does this sound like something you are interested in?

a. **YES**- That's great! Now we'll go ahead with the screening process to see if you qualify.

b. **NO**- Can I ask why you are not interested? – *indicate the reason not interested on Screening Tab in DB under "pre-screening notes/reason failed" and* *<u>check the pre-screen fail button</u>.*

If they decide they are not interested: Get as much info as you can (i.e. name, address, phone, DOB) and ask if they want to be contacted for future research. If they do not want future contact, delete their contact information from the Database.

PRESCREENING (EXCLUSIONARY CRITERIA) QUESTIONS

Administer "Pre-Screen Questionnaire."

Open the Screening Questionnaire Button.

Ask about physical activity and study participation.

If they are excluded based on the previous questions: Get as much info as you can (i.e. name, address, phone, DOB) and ask if they want to be contacted for future research. If they do not want future contact, delete their *contact information* from the Database.

If they respond negatively to the above questions and have been excluded:

> *Thank you for your interest Mr./Mrs.___________________, unfortunately you do not meet the initial criteria for our study. We have set criteria that we must maintain for all our participants and your responses did not fall within the guidelines we have determined for this study.*
>
> *Although you do not meet the guidelines for the current study, with your permission we would like to keep your information on file in the event that our criteria for this project changes or you qualify for another study within our laboratory.*

If at any time during the pre-screening phone call they decline to participate, ask them why they are no longer interested and only when appropriate gently encourage them to reconsider but DO NOT PUSH.

- ✓ *Always leave them with an opening to call us back if they reconsider their decision not to join us.*
- ✓ *Always ask permission to keep their information on file in the event that our criteria for this project changes or they qualify for another study within our laboratory.*

Regardless of the outcome of the call, always thank them.

- **If they answer yes to all of the above questions, they qualify and you may continue the screening.**

These next questions have to do with mental status and memory. These are questions we ask everyone and we just ask that you do the best you can. – Open tab in Database and administer TICS-M questionnaire.

- **If they score below "21" on TICS-M, they have been excluded:**

Thank you for your interest Mr./Mrs.___________________, unfortunately your answers did not fall within the range of acceptable responses and we are sorry you do not qualify for participation at this time. Your answers to the previous questions may suggest that you have potential difficulties remembering things. If this is of concern to you, we suggest that you contact your personal physician to determine whether a memory screening is necessary.

Although you do not meet the guidelines for the current study, with your permission we would like to keep your information on file in the event that our criteria for this project changes or you qualify for another study within our laboratory.

If at any time during the pre-screening phone call they decline to participate, ask them why they are no longer interested and only when appropriate gently encourage them to reconsider but DO NOT PUSH.

- ✓ *Always leave them with an opening to call us back if they reconsider their decision not to join us.*
- ✓ *Always ask permission to keep their information on file in the event that our criteria for this project changes or they qualify for another study within our department*

Regardless of the outcome of the call, always thank them.

- **If they score above "21" on TICS-M they qualify and you may continue the screening.**

These next questions have to do with your outlook on life. Your responses to these questions will not exclude you from participation, so we ask that you answer honestly. – Open tab in Database and administer GDS questionnaire.

We are finished with the pre-screening questionnaire and based on your responses you qualify for the study.

Great. Thanks (name). There are two more things we need to do if you have a few more minutes.

First I'd like to get some information from you regarding your Medical History. This is a fairly lengthy questionnaire however the answers are primarily "Yes or No" so it goes quickly.

If you have the time, let's start the Medical History Questionnaire.

- *Administer Personal Medical History Questionnaire*

OK, we have completed the Pre-Screening Interview portion for the program. I would like to schedule you for the Mock MRI appointment at the Beckman Institute. At this appointment, you will be placed in a mock version of the MRI machine so that we can determine if you will be able to do the actual MRI. You will be paid $10 for this appointment, which should take approximately one hour. (That is in addition to the payments I mentioned earlier for the subsequent testing.)

Open scheduling Calendar and schedule appointment.

I will be sending you a packet of information that will include the following including detailed instructions about everything that is included:

- Information and a reminder about the appointment we just scheduled;
- A campus map showing the locations of Beckman Institute and Freer Hall;
- Two copies of the Informed Consent. This is a document that discusses your role in this project, along with the risks and benefits associated with participation. This is a copy for you to keep for your records. I'll ask you to read it carefully and you will sign one to send back to us.
- A form for you to sign that will go to your physician allowing our office to send information about the study which he or she use to determine whether it is safe for you to participate in this exercise program.
- Envelopes for you to mail these two signed documents back to us separately.

After it has been determined that you passed the Mock MRI, we will hold an Informational Meeting for you and other study participants and I'll provide more information about this meeting at a later time.

Do you have any questions at this time? Thank you for your time today.

EMAIL RESPONSE

Thank you for your interest in the FAST project (Fit & Active Seniors Trial) at the University of Illinois at Urbana-Champaign funded by the National Institute on Aging and Abbott Nutrition. This email provides more information about the program. If you would like to continue the screening process, it takes about 30 minutes to ask you some additional questions, which we will do by phone. We have had a great response to our advertising and it may take me a day or two to get back to you. **Please let me know the best time to reach you by phone.**

We are examining the effects of physical activity training on how you process your thoughts and how your brain functions. We are looking for adults between 60 – 79 years of age who do not exercise on a regular basis to participate in a free six-month physical activity program at the University of Illinois at Urbana-Champaign.

If you qualify for this project, you would be *randomly assigned* to one of two activity groups:

- A group that focuses on stretching, strengthening, and stability work;
- A group that focuses on dancing such as English Country or Contra Dancing;
- A group that focuses on aerobic training in the form of walking; or
- A group that focuses on aerobic training in the form of walking while also taking a nutritional supplement that has been developed specifically for this study.

The groups are supervised by trained exercise specialists and meet three times a week for six months beginning [date] – Mondays, Wednesdays, and Fridays at 6:45 a.m. to 7:45 a.m. at different locations on the UI campus. Parking will be provided at no cost to you for all project-related visits to campus.

Prior to placement in one of the above activity groups, all participants must complete research-based testing including:

- Neuropsychological testing focusing on tasks related to memory, attention and decision-making
- Blood analysis
- Virtual Street crossing – walking on a self-propelled treadmill with "virtual" traffic visible to you on screens in front of the treadmill
- MRI of the brain
- Treadmill test
- Daily activity monitoring with a device similar to a pedometer
- Functional fitness tasks
- Completion of a battery of questionnaires

More information about these assessments will be provided.
All participants must:

- Be 60-79 years old
- Be able to provide physician's release to exercise, which we will facilitate
- Not exercise regularly more than two times per week
- Be able to walk on a treadmill
- Be able to complete a MRI of the brain
- Be right handed
- Be English speaking
- Be able to complete written questionnaires

- Intend to be in the local area during the study period with no absences of more than three weeks at a time

If you believe you meet all these requirements, please respond to this email and let me know when we can reach you to determine if you do qualify for this project. Thank you for your interest in FAST.

ACTIVE Adults *Recruitment procedures*

Initial phone call prescreening protocol

INTRODUCTION:

Hello. This is _________________ with the University of Illinois, Department of Kinesiology and Community Health. I'm returning your call about the Physical Activity study. [*exchange basic pleasantries – How are you today?]*

I would like to tell you about the study and if you are still interested, I will need to ask you some questions to make sure that you are eligible. Do you have time to talk right now?

a. **NO**—When would be a good time for me to give you a call back? – *indicate in DB when to call back.*

b. **YES**—Ok, great. [continue to contact information and demographics]

If they are not home leave the following message: Hello. This is _________________ with the University of Illinois, Department of Kinesiology and Community Health. I'm returning your call about the research project involving a physical activity study. Please feel free to call us again at (217)333-2427. Thank you.

CONTACT INFORMATION AND DEMOGRAPHICS:

Before we go too much further, may I have your name and some basic contact information?

– complete in DB under Contact information tab.

I would also like to get some basic demographic and general health information. This information is kept in a secure database in our lab and if there are any questions that you prefer not to answer, you don't have to. This information will help us verify that our advertising has reached a diverse representation of our area.

– complete in DB under Screening tab. [Continue through the questions about health and then to the exclusion questions.]

Thank you. Now let me tell you about the program.

DESCRIPTION OF PROGRAM:

The purpose of this study is to determine the effects of regular physical activity on how you process your thoughts and how your brain functions. We are looking for adults between 60 – 79 years of age who regularly exercise to participate in this study.

Everyone must be able to provide their physician's consent for them to be involved in the testing. We will facilitate this process.

As a requirement of the study, we will be asking you to perform some **psychological and fitness tests that include:**

- **A series of neuropsychological tests.** These are both computer-based and paper and pencil tests that will examine different aspects of your attention, memory and decision-making. These take place at the Beckman Institute on the UI campus.
- **Street crossing test.** During this assessment you will be asked to walk on a self-propelled treadmill while crossing a virtual street that will be presented on a screen in front of you. This test will take place at the Illinois Simulator Lab on the UI campus.
- **A functional MRI of the brain.** For this test, you will perform a series of question and answer tasks while we are collecting images of your brain at work. The amount of time you will spend in the MRI machine will be approximately 90 minutes. We will ask you to attend a "Mock MRI" prior to this to make sure that you will be able to tolerate the actual MRI in terms of comfort and claustrophobic issues. Although this is not a full-body MRI and these are scans of your brain only, you are placed in the magnet to approximately your waist and we will need to evaluate your tolerance for this test.
- **A graded exercise test.** This is a walking test on a treadmill. We will ask you to walk at a comfortable speed and we will adjust the grade (or the incline) of the treadmill to make it more challenging. You will wear a mask over your mouth and nose so that we can measure the air you use while exercising. You will walk until you feel you cannot walk any more.

All of these tests will take place on the UI campus. There are also a few things that we will ask you to do at home:

- We will ask you to wear an **accelerometer** for seven days. This is a device very much like a pedometer (but it records all activity not just your steps); you wear it on a strap around your waist.
- We will also ask you **complete a series of paper-and-pencil questionnaires** that you can do at home.

You will be paid in the following manner for completing these assessments that I just mentioned:

- Neuropsychological assessments– 1, 2hour appointment. Total $20
- Street crossing assessment– 1, 2hour appointment. Total $20

- fMRI – 2 hours of your time. Total $40
- Treadmill test – 1 hour. Total $20
-

So that's $100 for testing. You also receive $10 for the Mock MRI. And another reminder, there will be no cost to you for parking as well.

The benefits for you will be provided with feedback on all of your fitness assessments as well as making a contribution to the science and understanding of physical activity and the aging process.

Does this sound like something you are interested in?

a. **YES**- That's great! Now we'll go ahead with the screening process to see if you qualify.

b. **NO**- Can I ask why you are not interested? – *indicate the reason not interested on Screening Tab in DB under "pre-screening notes/reason failed" and check the pre-screen fail button.*

If they decide they are not interested: Get as much info as you can (i.e. name, address, phone, DOB) and ask if they want to be contacted for future research. If they do not want future contact, delete their contact information from the Database.

PRESCREENING (EXCLUSIONARY CRITERIA) QUESTIONS

[ENTERED INTO DATABASE]

Administer "Pre-Screen Questionnaire."

Open the Screening Questionnaire Button.

Ask about physical activity and study participation.

If they are excluded based on the previous questions: Get as much info as you can (i.e. name, address, phone, DOB) and ask if they want to be contacted for future research. If they do not want future contact, delete their *contact information* from the Database.

If they respond negatively to the above questions and have been excluded:

Thank you for your interest Mr./Mrs.__________________, unfortunately you do not meet the initial criteria for our study. We have set criteria that we must maintain for all our participants and your responses did not fall within the guidelines we have determined for this study.

Although you do not meet the guidelines for the current study, with your permission we would like to keep your information on file in the event that our criteria for this project changes or you qualify for another study within our laboratory.

If at any time during the pre-screening phone call they decline to participate, ask them why they are no longer interested and only when appropriate gently encourage them to reconsider but DO NOT PUSH.

- ✓ *Always leave them with an opening to call us back if they reconsider their decision not to join us.*
- ✓ *Always ask permission to keep their information on file in the event that our criteria for this project changes or they qualify for another study within our laboratory.*

Regardless of the outcome of the call, always thank them.

- **If they answer yes to all of the above questions, they qualify and you may continue the screening.**

These next questions have to do with mental status and memory. These are questions we ask everyone and we just ask that you do the best you can. – Open tab in Database and administer TICS-M questionnaire.

- **If they score below "21" on TICS-M, they have been excluded:**

Thank you for your interest Mr./Mrs.____________________, unfortunately your answers did not fall within the range of acceptable responses and we are sorry you do not qualify for participation at this time. Your answers to the previous questions may suggest that you have potential difficulties remembering things. If this is of concern to you, we suggest that you contact your personal physician to determine whether a memory screening is necessary.

Although you do not meet the guidelines for the current study, with your permission we would like to keep your information on file in the event that our criteria for this project changes or you qualify for another study within our laboratory.

If at any time during the pre-screening phone call they decline to participate, ask them why they are no longer interested and only when appropriate gently encourage them to reconsider but <u>DO NOT PUSH</u>.

- ✓ *Always leave them with an opening to call us back if they reconsider their decision not to join us.*
- ✓ *Always ask permission to keep their information on file in the event that our criteria for this project changes or they qualify for another study within our department*

Regardless of the outcome of the call, always thank them.

- **If they score above "21" on TICS-M they qualify and you may continue the screening.**

These next questions have to do with your outlook on life. Your responses to these questions will not exclude you from participation, so we ask that you answer honestly. – Open tab in Database and administer GDS questionnaire.

We are finished with the pre-screening questionnaire and based on your responses you qualify for the study.

Great. Thanks (name). There are two more things we need to do if you have a few more minutes.

First I'd like to get some information from you regarding your Medical History. This is a fairly lengthy questionnaire however the answers are primarily "Yes or No" so it goes quickly.

If you have the time, let's start the Medical History Questionnaire.

- *Administer Personal Medical History Questionnaire*

OK, we have completed the Pre-Screening Interview portion for the project. I would like to schedule you for the Mock MRI appointment at the Beckman Institute. At this appointment, you will be placed in a mock version of the MRI machine so that we can determine if you will be able to do the actual MRI. You will be paid $10 for this appointment, which should take approximately one hour. (That is in addition to the payments I mentioned earlier for the subsequent testing.)

Open scheduling Calendar and schedule appointment.

I will be sending you a packet of information that will include the following including detailed instructions about everything that is included:

- Information and a reminder about the appointment we just scheduled;
- A campus map showing the locations of Beckman Institute and Freer Hall;
- Two copies of the Informed Consent. This is a document that discusses your role in this project, along with the risks and benefits associated with participation. This is a copy for you to keep for your records. I'll ask you to read it carefully and you will sign one to send back to us.
- A form for you to sign that will go to your physician allowing our office to send information about the study which he or she use to determine whether it is safe for you to participate in this exercise program.
- Envelopes for you to mail these two signed documents back to us separately.

After it has been determined that you passed the Mock MRI, we will hold an Informational Meeting for you and other study participants and I'll provide more information about this meeting at a later time.

Do you have any questions at this time? Thank you for your time today.

ACTIVE Adults EMAIL RESPONSE

Thank you for your interest in the physical activity study. This email provides more information about the program. If you would like to continue the screening process, it takes about 30 minutes to ask you some additional questions, which we will do by phone. We have had a great response to our advertising and it may take me a day or two to get back to you.

Please let me know the best time to reach you by phone.

We are examining the effects of regular physical activity participation on health and well-being. We are looking for adults between 60 – 79 years of age who exercise on a regular basis to participate in a research study at the University of Illinois at Urbana-Champaign.
All participants must complete research-based testing including:

- Neuropsychological testing focusing on tasks related to memory, attention and decision-making
- Street crossing – walking on a self-propelled treadmill with "virtual" traffic visible to you on MRI of the brain
- Treadmill test
- Daily activity monitoring with a device similar to a pedometer
- Completion of questionnaires

More information about these assessments will be provided.
All participants must:

- Be 60-79 years old
- Be able to provide physician's release to exercise, which we will facilitate
- Be able to walk on a treadmill
- Be able to complete a MRI of the brain
- Be right handed
- Be English speaking
- Be able to complete written questionnaires

If you believe you meet all these requirements, please respond to this email and let me know when we can reach you to determine if you do qualify for this project.
Thank you for your interest in the physical activity study.

11. Inclusion and Exclusion Criteria-Low Active Adults

Inclusion	Exclusion
60-79 years of age	Below 60 years of age or above 80 years of age at beginning of intervention
Low-active: zero to two days of physical activity (> 30 minutes per day) per week in previous six months.	Self-reported regular physical activity of more than 2 times per week in last six months.
Personal physician's examination and/or consent to participate in testing and exercise intervention	Non-consent of physician
Successful completion of graded exercise test without evidence of cardiac abnormalities or responses which are likely to be exacerbated by exercise. This decision will be made by the attending cardiologist.	Evidence of abnormal cardiac responses or conditions during graded exercise testing
Adequate responses to the Telephone Interview of Cognitive Status (TICS-M) questionnaire (>21)	Inadequate responses to the Telephone Interview of Cognitive Status (TICS-M) questionnaire (<20)
Corrected (near and far) acuity of 20/40 or better in both eyes and no diagnosis of color-blindedness	Uncorrectable (near and far) acuity of greater than 20/40 in either eye and/or color-blindness
Initial depression score on GDS-15 below clinical level (> 10)	Depression score on GDS-15 indicative of clinical depression (≤ 10)
No presence of implanted devices or metallic bodies above the waist	Presence of any implanted devices above the waist i.e. cardiac pacemaker or auto-defibrillators; neural pacemaker; aneurysm clips in the CNS; cochlear implant; metallic bodies in the eye or CNS; any form of wires or metal devices that may concentrate radiofrequency fields
No history of brain surgery that involved removal of brain tissue	History of brain surgery that involved removal of brain tissue
Right-handed	Left-handed
No history of Stroke of TIA	History of Stroke or TIA
Intention to remain in the local area for the duration of the intervention or testing period	Intent to move or be away from the area for an extended period of time (i.e. > 3 weeks) during the intervention or

	testing period
English fluency	Inability to communicate effectively in English
Absence of chronic inflammation	Individuals with chronic inflammation i.e. IBS, lupus etc.

Inclusion and Exclusion Criteria-ACTIVE Adults

Inclusion	Exclusion
60-79 years of age	Below 60 years of age or above 80 years of age
High active: four days of aerobic physical activity (>30 minutes per day) per week in previous six months.	Self-reported regular physical activity of more less than 4 times per week in last six months.
Personal physician's examination and/or consent to participate in testing	Non-consent of physician
Successful completion of graded exercise test without evidence of cardiac abnormalities or responses which are likely to be exacerbated by exercise. This decision will be made by the attending cardiologist.	Evidence of abnormal cardiac responses or conditions during graded exercise testing
Adequate responses to the Telephone Interview of Cognitive Status (TICS-M) questionnaire (>21)	Inadequate responses to the Telephone Interview of Cognitive Status (TICS-M) questionnaire (<20)
Corrected (near and far) acuity of 20/40 or better in both eyes and no diagnosis of color-blindedness	Uncorrectable (near and far) acuity of greater than 20/40 in either eye and/or color-blindness
Initial depression score on GDS-15 below clinical level (> 10)	Depression score on GDS-15 indicative of clinical depression (≤ 10)
No presence of implanted devices or metallic bodies above the waist	Presence of any implanted devices above the waist i.e. cardiac pacemaker or auto-defibrillators; neural pacemaker; aneurysm clips in the CNS; cochlear implant; metallic bodies in the eye or CNS; any form of wires or metal devices that may concentrate radiofrequency fields
No history of brain surgery that involved removal of brain tissue	History of brain surgery that involved removal of brain tissue

No history of Stroke of TIA	History of Stroke or TIA
Right-handed	Left-handed
English fluency	Inability to communicate effectively in English

12. Research Procedures

These procedures are illustrated in the Participant Flowchart found at the end of this section. Healthy, sedentary older adults (n=250) and healthy, ACTIVE adults (n=50) will be recruited from the Champaign-Urbana area by a variety of advertising methods. This section addresses the larger group of sedentary adults. At the end of this section, procedures for the ACTIVE group will be presented.

Potential participants will be asked to contact study personnel and complete all screening procedures including a telephone interview in which contact information, physical activity behavior, and demographic information will be obtained. The research will then be described in detail to the participant and if he or she expresses continued interest, the Telephone Interview of Cognitive Status (TICS-M) will be administered. If the participant achieves an acceptable score (>21), pre-screening will be continued with the completion of the medical history. Medical release and two copies of the Informed Consent will be mailed to the participant with pre-stamped return envelopes included. The participant will be scheduled for a Mock MRI, to ensure successful completion of the future MRI, and additional cognitive screening to take place in a one-hour appointment at the Beckman Institute. If the participant successfully passes all criteria at this appointment, the medical release process will be initiated by EPL staff (i.e. faxing). If the participant is not able to complete the Mock MRI successfully, the attending Beckman staff member will inform him/her that they do not quailify for the study due to their inability to tolerate the actual fMRI environment. For those who successfully complete the Mock, they will continue with the MMSE screening questions. Those individuals who do not pass this screening task will be informed that they do not qualify and be given a letter of explanation.

Successfully screened individuals will be scheduled to attend an informational meeting to be held at Freer Hall. Following a presentation of the goals and procedures associated with this project, participants will be scheduled for baseline blood draw and provided with a copy of the Informed Consent associated with this lab visit. They will be also scheduled for the neuropsychological testing (described below); they will receive a packet of psychosocial questionnaires to complete at the meeting and another to complete on their own time at home and return at a subsequent Freer Hall appointment. The medical history (taken earlier by phone during screening) will be verified and signed by the participant. This information will be used for subsequent analyses (i.e. as covariates and predictors of outcomes, frequency analysis) and will not be identifiable within the data analysis file. Additionally, the medical history information may be presented in the form of sub-group frequencies or overall study

statistics to illustrate the percentage of people who may have co-morbitities in particular regions or groups. Participants will receive an accelerometer to wear for seven days as well as materials for mailing back the monitor. This device records all physical activity during waking hours.

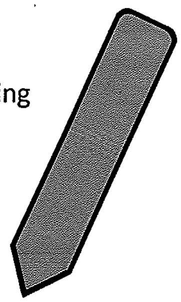

After medical release is received by the EPL, participants will be phoned to schedule the MRI, Graded Exercise Test and virtual street crossing appointments.

At the blood draw visit, which will take place in Dr. Jeffery A. Woods laboratory (Exercise Immunology Research Laboratory) in Freer Hall, participants will be asked to sign the informed consent related to the blood. At this visit, participants will provide a blood sample for CRP, IL-6, and BDNF analyses. Standard antecubital venipuncture will be performed by a trained phlebotimst using sterile technique. The maximal amount of blood drawn during any single sampling will be no more than 40mL. The amount of blood collected is much less than that given during a standard Red Cross blood donation (~500mL). In each case, the blood draw site will be bandaged and care instructions will be provided to the participant. Also during this appointment, participants will complete a small questionare packet (Packet C) and receive a light meal (i.e., juice and breakfast bar) following the blood draw. In total, the collection of the blood and completion of the questionnaire should take approximately 30 minutes.

Neuropsychological assessments will take place in one two-hour appointment held at the Beckman Institute. Assessments are both computer-based and paper-and-pencil based tasks that will include assessments of cognitive functions including: reaction time, task-switching, spatial orientation, planning, response inhibition, and working memory .

The street crossing assessment will take place in one two-hour appointment held at the Illinois Simulation Laboratory. Participants will be asked to walk on a self-propelled treadmill at a comfortable walking speed. This treadmill is surrounded by several screens in which a street scene with "virtual" moving traffic and a crosswalk will be visible to the participant as they are walking. They will be instructed to safely cross the "virtual" street by walking on the treadmill as quickly as possible. During some parts of this task, they will be asked to talk on a cell phone while still continuing to cross the street safely. This is a real-world assessment of multi-tasking.

The neuroimaging (MRI) session will last approximately two hours and will include completion of several tasks. Task performance, which occurs during MRI recording, will take no longer than 1 hour and 15 minutes, with the other 45 minutes being devoted to obtaining structural scans, system set-up, and subject familiarization with the tasks. The MRI recording sessions will occur within two to three

weeks before and after the intervention. This appointment will not occur on a day in which the participants have exercised.

Aerobic endurance capacity will be assessed on a motor-driven treadmill by employing a modified Balke protocol (graded exercise test -- GXT). The protocol involves walking at a self-selected pace with increasing grade increments of 2-3% every 2 minutes. Measurements of oxygen uptake, heart rate and blood pressure will be continuously monitored. Oxygen uptake (VO2) will be measured from expired air samples taken at 30-second intervals until a peak VO2 (the highest VO2) is attained; test termination is determined by symptom limitation, volitional exhaustion, and/or attainment of VO2 peak as per ACSM guidelines. The attending physician will have access to the participant's medical history (collected during screening and verified by the participant at the informational meeting) and will use this to ensure participant's safety during the test and be prepared with medical information and emergency contact information in event of an adverse reaction during the test. Graduate research assistants will also monitor and supervise all aspects of the graded exercise testing. The medical personnel will be responsible for interpretation of exercise test responses regarding ischemic coronary artery disease. Further measures of physical function will be assessed during the first week of the intervention with the Functional Fitness Test, a battery of simple tasks designed to determine physical function in older adults.

Assessments will be repeated at the end of the intervention (six-months/m6).

Following completion of the baseline testing mentioned above, one fourth of the participants will be randomly assigned to to the aerobic-cognitive combination dance group; one fourth will be assigned to a non-aerobic stretching, strengthening, and stability control group; one fourth will be assigned to the walking group; and one fourth will be assigned to the walking plus nutrtion group. Participants will attend a six-month supervised and progressive physical activity program that will meet three times per week. Program descriptions are below:

- **Aerobic-Cognitive Combination Dance Group:** This condition is designed to improve physical fitness as well specific cognitive functioning tasks through the combination of aerobic dance movement and the cognitive challenge of learning sequential dance steps and routines.

  The dance group program will be conducted three times per week (Monday, Wednesday, Friday) for six months. Sessions will be conducted in an appropriate dance space and will be instructed by experienced dance instructor(s). Each session will also have a trained exercise specialist

onsite. As the individuals in the groups will be non-active at the beginnning of the program, the dance group will start with easier choreographed dance combinations and progress to more challenging combinations over the course of the six month program. The dance combinations learned will include varities of ballroom, latin, square-dancing, polka, line-dancing, swing, folk, etc. The aerobic dancing choreography will include both partnered and non-partnered dancing. Since recruitment will most likely have an uneven gender representation, during the partnered dancing, all participants will learn both gender roles to maximize the cognitive challenge tasks involved.

- **Walking Group:** This condition is designed to increase physical fitness through brisk walking. Research staff will conduct supervised walking sessions three times per week. Frequent assessment of heart rate (both by palpation and Polar Heart Rate Monitors) and rating of perceived exertion will ensure that the participants' exercise intensity is performed at the prescribed level. Exercise logs will be completed after each exercise session noting frequency, intensity (RPE) and enjoyment levels associated with that session.

- **Walking Plus Nutritional Supplement Group:** This condition is designed to increase physical fitness through brisk walking as well as positively influence health through the administration of a nutrional supplement. Research staff will conduct supervised walking sessions three times per week. Frequent assessment of heart rate (both by palpation and Polar Heart Rate Monitors) and rating of perceived exertion will ensure that the exercise intensities are performed at the prescribed level. Exercise logs will be completed after each exercise session noting frequency, intensity (RPE) and enjoyment levels associated with that session. In addition to participating in the prescribed exercise, participants will also be instructed to consume a nutritional supplement daily. Research related to such supplements has suggested that dietary interventions may reduce the risk or delay the onset of age-associated cognitive decline and neurodegenerative diseases in humans. The supplement will be a nutritionally balanced formula, similar to Ensure® and is being developed specifically for this project in collaboration with Abbott Nutrition. It is to be delivered in a maltodextrin carrier with macro and micro-nutrients and will include: flavenoids (antioxidant polyphenol); resveratrol (antioxidant polyphenol); docosahexaenoic acid (anti-inflammatory); hydroxymethlbutrate (protein synthesis enhancement). The supplement will be provided to the participants and they will be asked to take this suppplement on a daily basis.

- **Stretching and Toning Control Group:** This group will serve as a non-aerobic control group against which to gauge the effects of the walking group and the dance group on neurocognitive, physical and psychosocial function. This group will meet with the same regularity as the other two aerobic exercise groups, will be led by an experienced exercise leader, and therefore will receive the same amount of attention as our treatment groups. The focus of the stretching and toning program is improving strength, stretching and stability for the whole body and is specially designed for individuals 60 years of age and older. Our program includes stretches, simple strength exercises, and basic balancing activities that are all age-appropriate and non-aerobic. Each stretch will be constant and gently held to a point of slight tension but not pain. Each stretch will be held for approximately 20-30 seconds. Our program will include stretches and simple strength exercises for all large muscle groups. The program includes functional balancing exercises. Each stretching and toning session will last for approximately 30-45 minutes and meet 3 times per week. Each session will be preceded and followed by 10-15 minutes of warm-up and cool-down exercises. Such a program is unlikely to result in significant increases in aerobic capacity.

  All strength and balance sessions will be conducted at facilities on the University of Illinois campus. The exercise classes will be conducted and supervised by trained exercise specialists. These sessions will take place in an exercise-specific studio or other appropriate indoor facilities. We have previously employed this tactic with success. Subjects will be instructed in how to monitor ratings of perceived exertion during the first few exercise sessions. Subjects will also receive their exercise logs and instructions for completing them. Exercise logs will be collected by the exercise leader at the end of each week.

During the first week of the intervention, attendance at an orientation will be required of all participants; detailed information about their program will be presented including a description of the physical activity program as well as the logs they will be completing. Individuals in both groups will be provided with a binder for maintaining exercise session logs used for attendance and tracking of exercise performance.

During the first week of participation all participants will complete the Functional Fitness assessment.

In addition to the baseline and six functional assessments and psychosocial questionnaires, Measures 11, 12, 13, 14, 15, and 16 will also be collected at the three-week time-point to assess changes in self-efficacy.

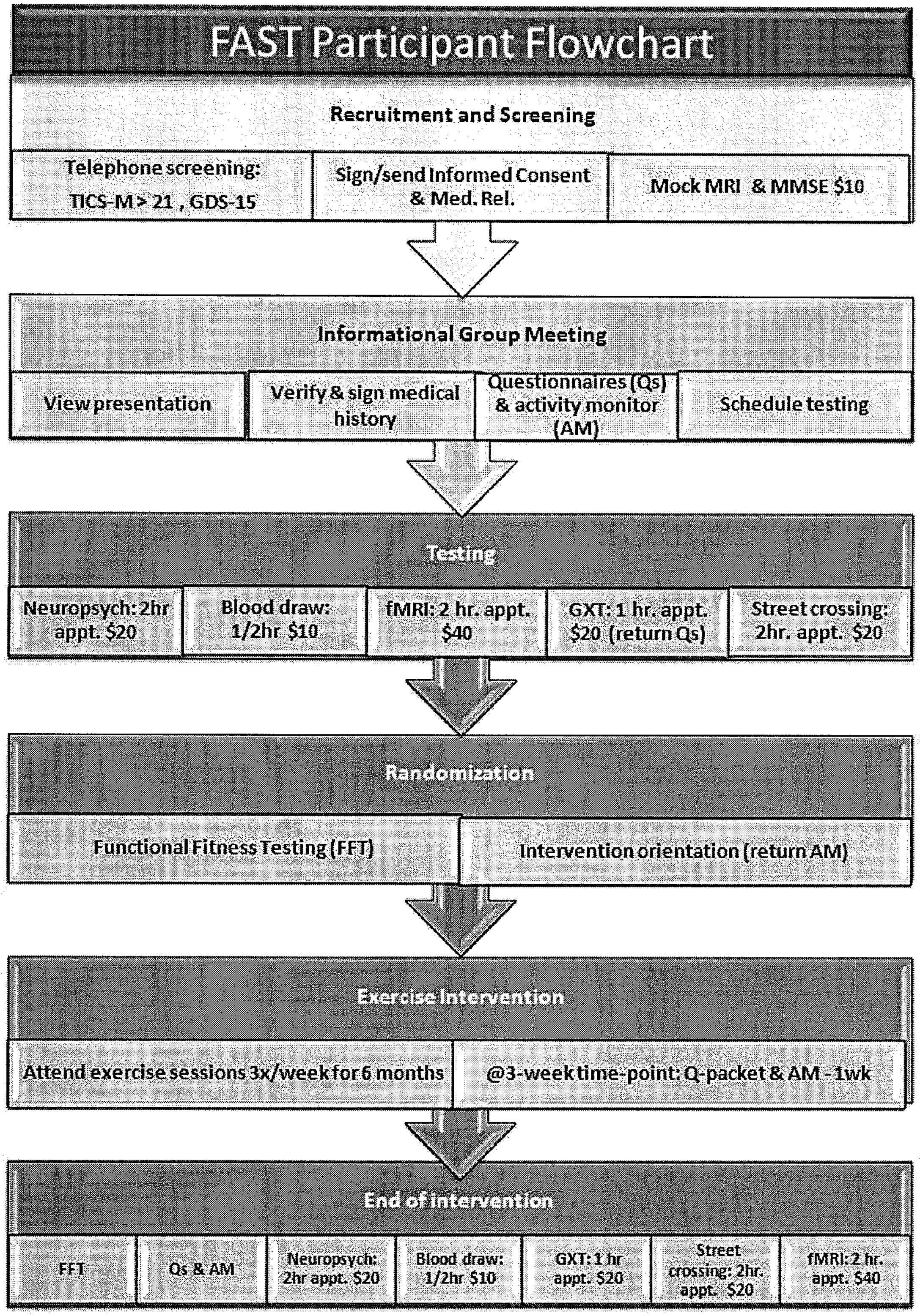
FAST Participant Flowchart
Recruitment and Screening
Telephone screening: TICS-M > 21 , GDS-15
Sign/send Informed Consent & Med. Rel.
Mock MRI & MMSE $10
Informational Group Meeting
View presentation
Verify & sign medical history
Questionnaires (Qs) & activity monitor (AM)
Schedule testing
Testing
Neuropsych: 2hr appt. $20
Blood draw: 1/2hr $10
fMRI: 2 hr. appt. $40
GXT: 1 hr. appt. $20 (return Qs)
Street crossing: 2hr. appt. $20
Randomization
Functional Fitness Testing (FFT)
Intervention orientation (return AM)
Exercise Intervention
Attend exercise sessions 3x/week for 6 months
@3-week time-point: Q-packet & AM - 1wk
End of intervention
FFT
Qs & AM
Neuropsych: 2hr appt. $20
Blood draw: 1/2hr $10
GXT: 1 hr appt. $20
Street crossing: 2hr. appt. $20
fMRI: 2 hr. appt. $40

ACTIVE Adults *Procedures*

Potential ACTIVE participants will be asked to contact study personnel and complete all screening procedures including a telephone interview in which contact information, physical activity behavior, and demographic information will be obtained. The research will then be described in detail to the participant and if he or she expresses continued interest, the Telephone Interview of Cognitive Status (TICS-M) will be administered. If the participant achieves an acceptable score (>21), pre-screening will be continued with the completion of the medical history. Medical release and two copies of the Informed Consent will be mailed to all older adult participants with pre-stamped return envelopes included, while young adults will complete these items at the informational meeting (to be addressed later). The participants will be scheduled for a Mock MRI, to ensure successful completion of the future MRI, and additional cognitive screening to take place in a one-hour appointment at the Beckman Institute. If the participant successfully passes all criteria at this appointment, the medical release process will be initiated by EPL staff (i.e. faxing). If participants are not able to complete the Mock MRI successfully, the attending Beckman staff member will inform them that they do not quailify for the study due to their inability to tolerate the actual fMRI environment. For those who successfully complete the Mock, they will continue with the MMSE screening questions. Those individuals who do not pass this screening task will be informed that they do not qualify and be given a letter of explanation.

Successfully screened older adult and young adult individuals will be scheduled to attend an informational meeting to be held at Freer Hall. Following a presentation of the goals and procedures associated with this project, participants will be scheduled for neuropsychological testing appointment and the street crossing appointment (described below); they will receive a packet of psychosocial questionnaires to complete at the meeting. In addition, participants will also receive an accelerometer to wear for seven days. This device records all physical activity during waking hours. Along with the monitior, participants will be given another questionaire to complete on their own time at home and return at a subsequent Freer Hall appointment. The medical history (taken earlier by phone during screening) will be verified and signed by the participant as well.

After medical release is received by the EPL, for all older adult participants, participants will be phoned to schedule, MRI and Graded Exercise Test appointments.

Neuropsychological assessments will take place in one two-hour appointment held at the Beckman Institute. Assessments are both computer-based and paper-and-pencil based tasks that will include assessments of cognitive functions including: reaction time, task-switching, spatial orientation, planning, response inhibition, and working memory .

The street crossing assessment will take place in one two-hour appointment held at the Illinois Simulator Laboratory. Participants will be asked to walk on a self-propelled treadmill at a comfortable walking speed. This treadmill is surrounded by several screens in which a street scene with "virtual" moving traffic and a crosswalk will be visible to the participant as they are walking. They will be instructed to safely cross the "virtual" street by walking on the treadmill as quickly as possible. During some parts of this task, they will be asked to talk on a cell phone while still continuing to cross the street safely. This is a real-world assessment of multi-tasking.

The neuroimaging (MRI) session will last approximately two hours and will include completion of several tasks. Task performance, which occurs during MRI recording, will take no longer than 1 hour and 15 minutes, with the other 45 minutes being devoted to obtaining structural scans, system set-up, and subject familiarization with the tasks. This appointment will not occur on a day in which the participants have exercised or participated in any fitness testing.

Aerobic endurance capacity will be assessed on a motor-driven treadmill by employing a modified Balke protocol (graded exercise test -- GXT). The protocol involves walking at a self-selected pace with increasing grade increments of 2-3% every 2 minutes. Measurements of oxygen uptake, heart rate and blood pressure will be continuously monitored. Oxygen uptake (VO2) will be measured from expired air samples taken at 30-second intervals until a peak VO2 (the highest VO2) is attained; test termination is determined by symptom limitation, volitional exhaustion, and/or attainment of VO2 peak as per ACSM guidelines. A physician and graduate research assistants will monitor and supervise all aspects of the graded exercise testing. The medical personnel will be responsible for interpretation of exercise test responses regarding ischemic coronary artery disease.

16. Measures

Prescreen:	Done over the phone when potential participant initiates interest in program.
Orientation Packet:	Verified at Informational Meeting.
A-Packet:	Given to participant at Informational Meeting to complete and return at end of meeting.
B-Packet	Given to participants to complete at home and returned at GXT appointment.
Week 3 Packet	Given to participants on the first day (Monday) of week 4 to complete before exercise and returned.

Administration Schedule			Baseline m0				m6		
Questionnaires[1]	Pre-Screen	Participant info.	A-Packet	B-Packet	C-Packet	Week 3	A-Packet	B-Packet	C-Packet
1. Pre-Screen Q. w/ GDS-15	✓						✓		
2. TICS-M	✓								
3. Personal Medical History	✓	✓[2]					✓[3]		
4. MMSE	✓[4]								
5. DEMO	✓								
6. Informed Consent-Umbrella		✓							
7. GLTEQ			✓				✓		
8. PASE			✓				✓		
9. SWLS			✓				✓		
10. HADS			✓				✓		
11. SE_Walk			✓			✓	✓		

[1] Abbreviated title can be found at upper right corner of questionnaire in Appendix.
[2] Administered over phone during pre-screen and verified and signed at Informational Meeting.
[3] Will be double-checked for new medical conditions.
[4] Administered at Beckman during Mock MRI/Neuropsychological appointment.

			m0-A	m0-B	m0-C	wk3	m6-A	m6-B	m6-C
12. LSE			✓			✓	✓		
13. GES			✓			✓	✓		
14. STQ			✓				✓		
15. FXNSE					✓	✓			
16. EXSE				✓		✓		✓	
17. BARSE					✓	✓			✓
18. SlfReg					✓				✓
19. PMI				✓				✓	
20. UCLA					✓				✓
21. CES-D					✓				✓
22. PSS					✓				✓
23. PACES				✓				✓	
24. FDI_FXN_abb				✓				✓	
25. MOEES				✓				✓	
26. LOT-R					✓				✓
27. BIF				✓				✓	
28. FLOUR					✓				✓
29. SPANE					✓				✓
30. SF-12				✓				✓	
31. FOF				✓				✓	
32. MCI				✓				✓	

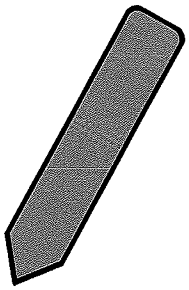

			m0	m0-B	m0-C	wk3	m6-A	m6-B	m6-C
33. RSE				✓				✓	
34. PSQI				✓				✓	
35. SPS				✓				✓	
36. SNI				✓				✓	
37. PSPP				✓				✓	
38. CRF	Administered at GXT at both time-points								

ACTIVE Adult Measures

Prescreen: Done over the phone when potential participant initiates interest in program.
Orientation Packet: Verified at Informational Meeting.
A-Packet: Given to participant at Informational Meeting to complete and return at end of meeting.
B-Packet Given to participants to complete at home and return at GXT.

Administration Schedule<br><br>Questionnaires[5]	Pre-Screen	Participant info.	A- packet	B-Packet
1. ACTIVE Pre-Screen Q. w/ GDS-15	✓			
2. TICS-M	✓			
3. Personal Medical History[6]	✓	✓		
4. MMSE	✓			
5. DEMO	✓			
6. Informed Consent-Umbrella		✓		
7. GLTEQ			✓	
8. PASE			✓	
9. SWLS			✓	
10. HADS			✓	
11. SE_Walk			✓	
12. LSE			✓	
13. GES			✓	
14. STQ			✓	

[5] Abbreviated title of questionnaire is found at upper right corner of questionnaire in Appendix.
[6] Administered over the phone during pre-screening and verified at Informational Meeting for Active Participants.

15. FXNSE				✓
16. EXSE				✓
17. BARSE				✓
18. SlfReg				✓
19. PMI				✓
20. UCLA				✓
21. CES-D				✓
22. PSS				✓
23. PACES				✓
24. FDI_FXN_abb				✓
25. MOEES				✓
26. LOT-R				✓
27. BIF				✓
28. FLOUR				✓
29. SPANE				✓
30. SF-12				✓
31. FOF				✓
32. MCI				✓
33. RSE				✓
34. PSQI				✓
35. SPS				✓
36. SNI				✓
37. PSPP				✓
38. CRF	Administered at GXT			

21A. Risks

The nutritional supplement will be administered in a matrix known to have very low allergic properties and will compounds known to have antioxidant, anti-inflammatory and protein synthesis enhancement properties.
